# Transient Laser‐Shocked Synthesis of Amorphous Layer‐Supported Metal Nanocrystals for Efficient Nitrate Reduction

**DOI:** 10.1002/adma.73764

**Published:** 2026-06-18

**Authors:** Weihua Guo, Jixun Zhang, Siwei Zhang, Yangbo Ma, Yun Song, Jianjun Su, Zihao Li, Yinger Xin, Qiang Zhang, Mingming He, Ruixuan Wang, Rui Xue, Shibo Xi, Ying Wang, Shenlong Zhao, Tao Yang, Zhengxiao Guo, Ben Zhong Tang, Ruquan Ye

**Affiliations:** ^1^ Department of Chemistry State Key Laboratory of Marine Pollution City University of Hong Kong Hong Kong China; ^2^ City University of Hong Kong Shenzhen Research Institute Shenzhen Guangdong China; ^3^ Department of Materials Science and Engineering City University of Hong Kong Hong Kong China; ^4^ Department of Chemistry and the Hong Kong Branch of Chinese National Engineering Research Center for Tissue Restoration and Reconstruction The Hong Kong University of Science and Technology Hong Kong China; ^5^ Department of Chemistry The University of Hong Kong Hong Kong China; ^6^ Institute of Chemical and Engineering Sciences A*STAR Singapore Singapore; ^7^ Department of Chemistry Chinese University of Hong Kong Hong Kong China; ^8^ CAS Key Laboratory of Nanosystem and Hierarchical Fabrication CAS Center for Excellence in Nanoscience National Center for Nanoscience and Technology Beijing China; ^9^ Guangdong Basic Research Center of Excellence for Aggregate Science School of Science and Engineering The Chinese University of Hong Kong Shenzhen (CUHK‐Shenzhen) Guangdong China

**Keywords:** amorphous/crystalline interfaces, interfacial engineering, laser‐shocked synthesis, metal support interaction, nitrate reduction reaction

## Abstract

Metal‐support interactions provide a powerful tool to tailor the catalytic activities of metallic catalysts. Amorphous materials can serve as an effective support matrix to form unique crystalline‐amorphous interfaces and modulate the electronic structure of active metals. However, robust synthetic strategies for precise structural control remain underdeveloped. Here, we report the laser‐shocked synthesis of heterostructures including bimetallic CuNi, CuFe, CuCo, and medium‐entropy CuFeCoNi heterostructures, where crystalline metal nanoparticles are anchored on amorphous hydroxide supports. The heterostructures are characterized by an interfacial electronic distribution that improves catalytic activities. With CuNi as an example for nitrate reduction reaction, the laser‐engineered heterophase CuNi achieves an NH_3_ production rate of 92.18 mg/h/mg_cat_ with 98.6% Faradaic efficiency (FE), substantially superior to standalone crystalline CuNi or amorphous CuNi hydroxide. The CuNi heterostructure maintains a stable FE(NH_3_) of ∼90% up to 80 h while improving current density from 75 to 120 mA/cm^2^ due to the robust amorphous layer and dynamic amorphous/crystalline reconstruction. In situ characterization and theoretical calculations reveal that the amorphous/crystalline interface regulates the balance between reactive hydrogen species and reaction intermediates, effectively suppressing the competing hydrogen evolution and promoting cascade nitrate‐to‐nitrite and nitrite‐to ammonia conversion. This work provides a general and viable strategy for producing high‐performance supported catalysts.

## Graphical abstract

A general, versatile laser‐shock synthesis process is reported to produce various bimetallic CuNi, CuFe, CuCo, and medium‐entropy CuFeCoNi heterostructures, in which crystalline metal nanoparticles are anchored to amorphous hydroxide supports. During nitrate reduction reaction, the heterostructures undergo dynamic amorphous/crystalline reconstruction and maintain the heterogeneous interfaces, demonstrating 98.6% nitrate‐to‐ammonia Faradaic efficiency with 80‐h stability.

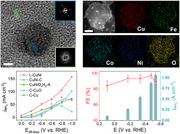

## 1 Introduction

Metal–support interactions (MSI) have emerged as a powerful strategy for modulating catalytic performance, owing to their ability to induce dynamic restructuring under redox and reaction conditions, as well as to fine‐tune adsorbate binding at active sites [1, 2, 3]. The design of heterogeneous catalysts has expanded beyond conventional nanoparticle–support systems to amorphous/crystalline heterostructures [1, 3, 4, 5]. These composites leverage synergistic effects between structurally distinct domains to significantly enhance electrocatalytic activity [6, 7, 8, 9, 10, 11]. The amorphous phases, characterized by randomly oriented and unsaturated coordination environments, serve as an effective support matrix that can modify the electronic structure of the active metal [9, 12, 13, 14, 15]. Furthermore, the crystalline–amorphous interface not only strengthens electronic coupling to optimize key parameters such as valence state and hydrogen adsorption energy, but also improves the stability of the catalyst under operating conditions [16, 17]. Such interfaces have been demonstrated to enhance reaction kinetics in processes such as the hydrogen evolution reaction and CO–CO coupling, where the dynamic nature of the amorphous phase under reaction conditions further contributes to the generation and stabilization of active sites [6, 12, 18, 19]. Thus, amorphous/crystalline heterostructures represent a promising avenue for designing robust and highly efficient electrocatalysts.

The synthesis of metal oxide‐supported nanoparticle catalysts can be achieved through various methods, including impregnation, hydrothermal/solvothermal synthesis, and chemical vapor deposition [1, 3, 4, 20, 21, 22, 23, 24, 25, 26]. However, conventional techniques often face challenges in controlling particle size, dispersion, and defect density. In this context, laser‐assisted synthesis emerges as a promising alternative, offering several distinct advantages [27, 28, 29, 30, 31, 32, 33, 34, 35]. This technique is typically performed in a liquid phase at ambient temperature and pressure, significantly simplifying the experimental setup. Its most defining feature is the ultra‐rapid heating and quenching (at rates of 10^6^ –10^9^ K/s), which effectively suppresses nanoparticle agglomeration and Ostwald ripening [36]. Compared to single‐element a/c systems [18], our laser‐assisted method enables production of alloy/hydroxide heterostructures from bimetal to medium‐entropy metals. Consequently, laser synthesis enables the creation of nanomaterials with highly dispersed, ultrasmall nanoparticles and an abundance of catalytically active defects, which are desired for high‐performance catalysts.

Based on the discussion, we herein report a laser‐assisted and scalable strategy for synthesizing highly active catalysts, with characteristics of crystalline nanoparticles supported on amorphous mixed metal hydroxide. We start with laser irradiation of a CuNi alloy precursor in water, resulting in the formation of crystalline CuNi nanoparticles anchored within an amorphous CuNi hydroxide support, denoted as L‐CuNi. This versatile method can be extended to produce a range of nanomaterials composed of crystalline alloys supported on amorphous hydroxide substrates, including bimetallic CuFe, CuCo, and medium‐entropy CuFeCoNi. Electron microscopy and spectroscopic analyses reveal a dual‐phase reaction mechanism during laser processing, leading to the concurrent formation of an amorphous hydroxide phase and crystalline alloy nanoparticles.

To examine the role of amorphous support, we use nitrate reduction reaction (NITRR) to produce ammonia [37, 38, 39, 40, 41, 42, 43], a reaction for remediating nitrate‐rich wastewater [44, 45, 46]. Achieving high NITRR efficiency is challenged by its complex reaction pathway, which requires a precise balance in the supply of active hydrogen (H_ads_) from water dissociation [47, 48, 49, 50, 51, 52, 53, 54, 55, 56]. Insufficient H_ads_ leads to incomplete reduction and nitrite accumulation, while excess H_ads_ favors the competing hydrogen evolution reaction [47, 57]. The resulting L‐CuNi catalyst achieves a remarkable NH_3_ yield rate of up to 65.2 mg h^−1^ mg_cat_^−1^ with high Faradaic efficiency (>80%). The catalyst exhibits outstanding stability, maintaining its activity and selectivity over 80 h of continuous operation. In situ Fourier transform infrared spectroscopy (FTIR) spectroscopy combined with density functional theory (DFT) calculations further elucidates the critical role of the amorphous oxide support in regulating the dynamic balance between H_ads_ and the key intermediate *NO_2_, thereby enhancing both the selectivity and durability of the reaction.

## 2 Results

### 2.1 Characterization of the Synthesis Mechanism of Laser Ablation Technology

**FIGURE 1:**
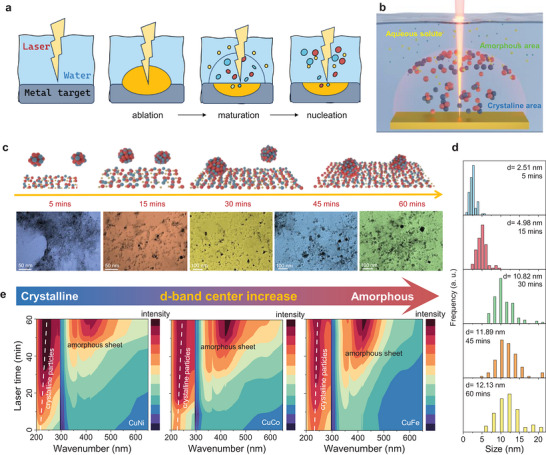
Characterization of the synthesis mechanism of laser ablation technology. (a) Schematic illustration of three steps in the process during laser ablation in liquid. (b) Synthesis mechanism of L‐CuNi. Red ball: Cu atom; blue ball: Ni atom; yellow ball: O atom; cyan ball: H atom; gold template: CuNi target material. (c) HR‐TEM images of L‐CuNi at different times during the process of laser ablation. (d) Particle size statistics in different laser times according to (c), the number of measured particles (N) for each time point is: 5 min, N = 101; 15 min, N = 104; 30 min, N = 121; 45 min, N = 115; 60 min, N = 114. Average diameters are indicated by dashed lines. (e) UV spectrum in different laser times of L‐CuNi, L‐CuCo, and L‐CuFe. Scale bar: 100 nm in (c). Red ball: Cu atom; blue ball: Ni atom; yellow ball: O atom; cyan ball: H atom; gold template: CuNi target material.

The synthesis of metal nanoparticles via laser ablation typically involves three key stages (Figure 1a). Initial laser shock leads to ablation of material at the metal target/water interfaces, forming a transient liquid phase that induces seed nucleation. This process produces a concentrated mixture of atoms and clusters. Subsequently, rapid evaporation driven by thermodynamic instability leads to frequent collisions and coalescence events, forming small nanoparticles, which are rapidly quenched by the surrounding fluid. Finally, under continued laser exposure, the nanoparticles undergo growth and maturation.

The specific formation pathway and resulting morphology depend strongly on the properties of the metal [58, 59, 60]. Non‐noble metals such as iron, cobalt, and nickel, which possess high *d*‐band centers, readily interact with oxygen and hydrogen atoms from the solvent. Coupled with rapid cooling, which restricts the time available for atomic rearrangement, this promotes the formation of amorphous nanosheets rather than crystalline structures. Hence, these surface nanosheets act as a “sacrificial layer,” protecting the underlying material from further oxidation in the liquid and facilitating the formation of metallic particles [61]. As illustrated in Figure 1b, near the periphery of the energy wave, copper and nickel atoms bond with oxygen and hydrogen atoms from the solution, leading to the formation of amorphous nanosheets. In contrast, atoms in the core region are shielded from direct contact with solvent‐derived oxygen, allowing the formation of a Cu–Ni alloy that subsequently solidifies into crystalline nanoparticles upon rapid cooling. The fundamental reason why the same rapid cooling process can lead to either amorphous or crystalline structures lies in the incorporation of oxygen and hydrogen atoms. Their presence introduces lattice distortion, and the slow diffusion kinetics under rapid quenching restricts atomic rearrangement into an ordered lattice [36, 62]. These combined effects promote amorphization in the peripheral regions, while the core region retains a crystalline structure due to the inert local environment.

We characterized the morphology of a bulk CuNi target immersed in deionized water after different durations of laser irradiation. Transmission electron microscope (TEM) images reveal that in the initial stage, small nanosheets and clusters are formed. As laser irradiation time increases, both the nanosheets and nanoparticles exhibit noticeable growth (Figure 1c). According to particle size statistics, the average diameter of the clusters increases from 2.5 to 10.8 nm after 30 min of irradiation. With further extension of the lasing time, the growth rate becomes slow, and the particle size stabilizes at ∼12.1 nm at 60 min (Figure 1d and Figure S1).

To better characterize the formation of amorphous and crystalline phases during the laser process, we monitored the growth of L‐CuNi using ultraviolet‐visible (UV–vis) spectroscopy (Figure 1e left). The optical responses reflect distinct plasmonic behaviors governed by nanoparticle morphology [63, 64, 65, 66]. Owing to the symmetry of spherical nanoparticles, their electron clouds resonate at a specific frequency, resulting in a single, sharp, and symmetric absorption peak around 230 nm. The absence of a surface plasmon resonance peak around 580 nm indicates that no free copper nanoparticles were formed. Furthermore, the characteristic absorption peak of the alloy is blue‐shifted to a shorter wavelength, which can be attributed to the small particle size [67]. This peak progressively red shifts with prolonged laser irradiation, which is a signature of nanoparticle growth, as the plasmon resonance is sensitive to particle size [64, 68]. In contrast, anisotropic nanosheets exhibit multiple absorption peaks due to their shape‐dependent plasmon modes: a strong and broad in‐plane dipole resonance peak, significantly red‐shifted to around 420 nm due to the extended lateral dimension. A weaker out‐of‐plane quadrupole resonance appears at shorter wavelengths of 350 nm due to electron oscillations along the thickness direction [63, 66]. Moreover, the steepness of the absorption edge is closely related to the crystallinity and defect density of the material. A sharper absorption edge indicates higher crystallinity and fewer structural defects, whereas a more extended tailing effect suggests greater structural disorder and a higher concentration of defect states [65]. As clearly observed in the results, the nanosheets exhibit significant absorption tailing, consistent with their amorphous nature. In contrast, the nanoparticles show a steep absorption edge, hinting at their high crystallinity.

To assess the generality of the laser‐driven synthesis for metallic nanomaterials, we substituted nickel with cobalt and iron, producing L‐CuCo and L‐CuFe. This experiment was designed to determine if the lasing process could yield analogous architecture. The UV‐Vis spectra of L‐CuCo and L‐CuFe exhibit characteristic peaks analogous to those of L‐CuNi, corresponding to both amorphous nanosheets and crystalline nanoparticles, indicating the successful synthesis of materials with identical structures and morphologies (Figure 1e). By comparing the UV–vis spectra of L‐CuFe, L‐CuCo, and L‐CuNi, we further observed that the peak intensity at around 420 cm^−1^, corresponding to nanosheets, follows the order Fe > Co > Ni, suggesting a decreasing tendency to form amorphous nanosheets in the sequence of Fe > Co > Ni. Conversely, the intensity of the nanoparticle‐related peak near 230 cm^−1^ increases in the order Fe < Co < Ni, indicating a growing trend toward the formation of crystalline nanoparticles. This behavior can be attributed to the fact that Fe possesses a higher *d*‐band center than Co and Ni, making it more prone to interact with oxygen and hydrogen species in water, thereby facilitating the formation of amorphous nanosheets [65]. In contrast, Ni, with the lowest *d*‐band center among the three, exhibits greater stability and thus promotes the crystallization of CuNi nanoparticles.

### 2.2 Catalyst Synthesis and Characterization

**FIGURE 2:**
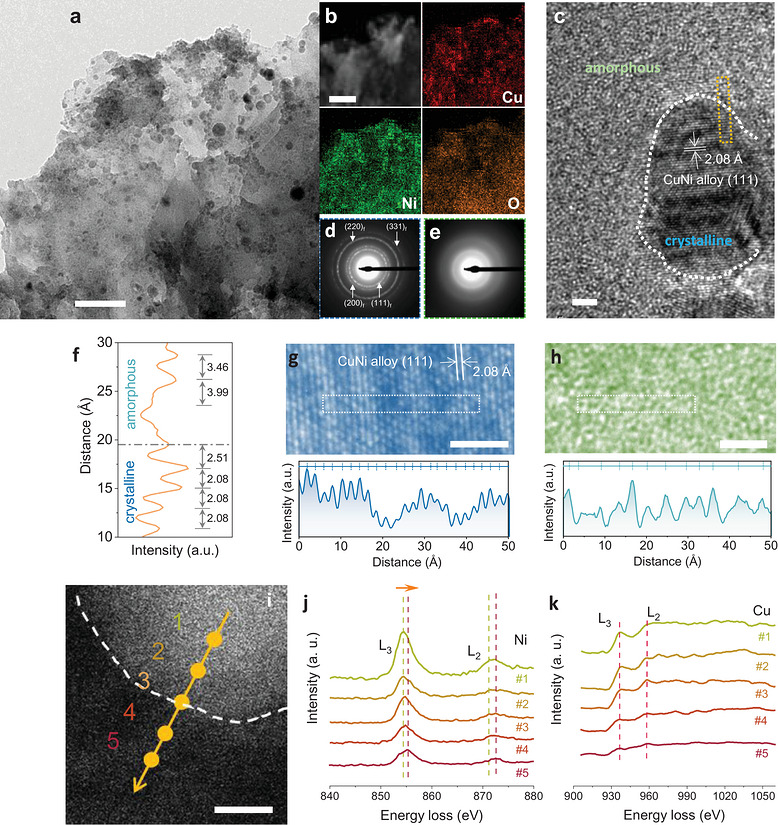
Morphology characterization of L‐CuNi. (a) TEM, (b) corresponding EDX mapping, and (c) HR‐TEM images of L‐CuNi. (d,e) SAED patterns of the crystalline zone and amorphous zone for L‐CuNi. (f)The line intensity profile acquired along the areas in (c). (g,h) High‐resolution HAADF‐STEM image of the crystalline zone and amorphous zone enlarged area in (c) and corresponding line intensity profile. (i) Scanning path in the STEM–HAADF image for EELS profile spectra extraction, with the labelled positions in (j,k). EELS spectra of Ni L‐edge (j) and Cu L‐edge (k) for different positions on L‐CuNi. Scale bar: 100 nm in (a and b), 2 nm in (c, g, h), and 5 nm in (i).

High‐resolution (HR) TEM was used to analyze the microstructure of L‐CuNi (Figure 2a–c; Figures S2‐S5). As shown in Figure 2a, the L‐CuNi exhibits ultrathin nanosheets with nanoparticles dispersed on the surface. The energy‐dispersive X‐ray (EDX) elemental mappings (Figure 2b) show that Cu, Ni, and O elements are uniformly distributed in the entire sample. Scanning transmission electron microscopy (STEM) images clearly reveal the distinct atomic features of the amorphous nanosheets and crystalline nanoparticles (Figure 2c). From the selected area electron diffraction (SAED) patterns (Figure 2d,e), the rings formed by the bright spots correspond to the crystal planes of CuNi, indicating it is crystalline, and the absence of bright rings confirms the amorphous state of the flocculent area. The above results confirm that the ultrathin nanosheets correspond to amorphous CuNiO_x_H_y_ and the nanoparticles correspond to crystalline CuNi. The intensity profiles along the amorphous/crystalline domains (Figure 2f) delineate the transition, revealing a locally widened interatomic distance of the crystalline CuNi near the interface. This could also be clearly seen in the surface intensity profile, showing a relatively consistent interatomic distance with changes in intensity. In stark contrast, the CuNiO_x_H_y_ nanosheets show a completely disordered surface; the intensity profile demonstrates random intensity and interatomic distance (Figure 2g,h).

To further understand the interface charge distribution, line‐scan electron energy loss spectroscopy (EELS) spectra across the crystal/amorphous interface were acquired (Figure 2i and Figures S6 and S7). In EELS valence analysis, the *L*‐edge energy shift of Ni provides a direct indication, as its oxidation state manifests as a consistent chemical shift. In contrast, for Cu, the transition from a closed‐shell (Cu^0^) to an open‐shell (Cu^2^^+^) configuration causes a dramatic change in L_3_ white‐line intensity, making its shape and strength a more accurate diagnostic [69, 70]. Consistent with this distinction, the Ni L‐edge spectra exhibit a positive chemical shift, indicating an increasing electron deficiency of Ni when transitioning from the crystalline particle across the interface into the amorphous region (Figure 2j) [69]. Meanwhile, the gradual intensification of the Cu L_3_ white‐line peak at the interface suggests the progression of Cu species from a metallic to an oxidized state (Figure 2k) [3, 70]. The EELS spectra result from interface #3 provide direct evidence of electron transfer from crystalline CuNi to the amorphous CuNiOxHy‐A support. This electron redistribution serves as a key regulatory mechanism for catalytic enhancement.

**FIGURE 3:**
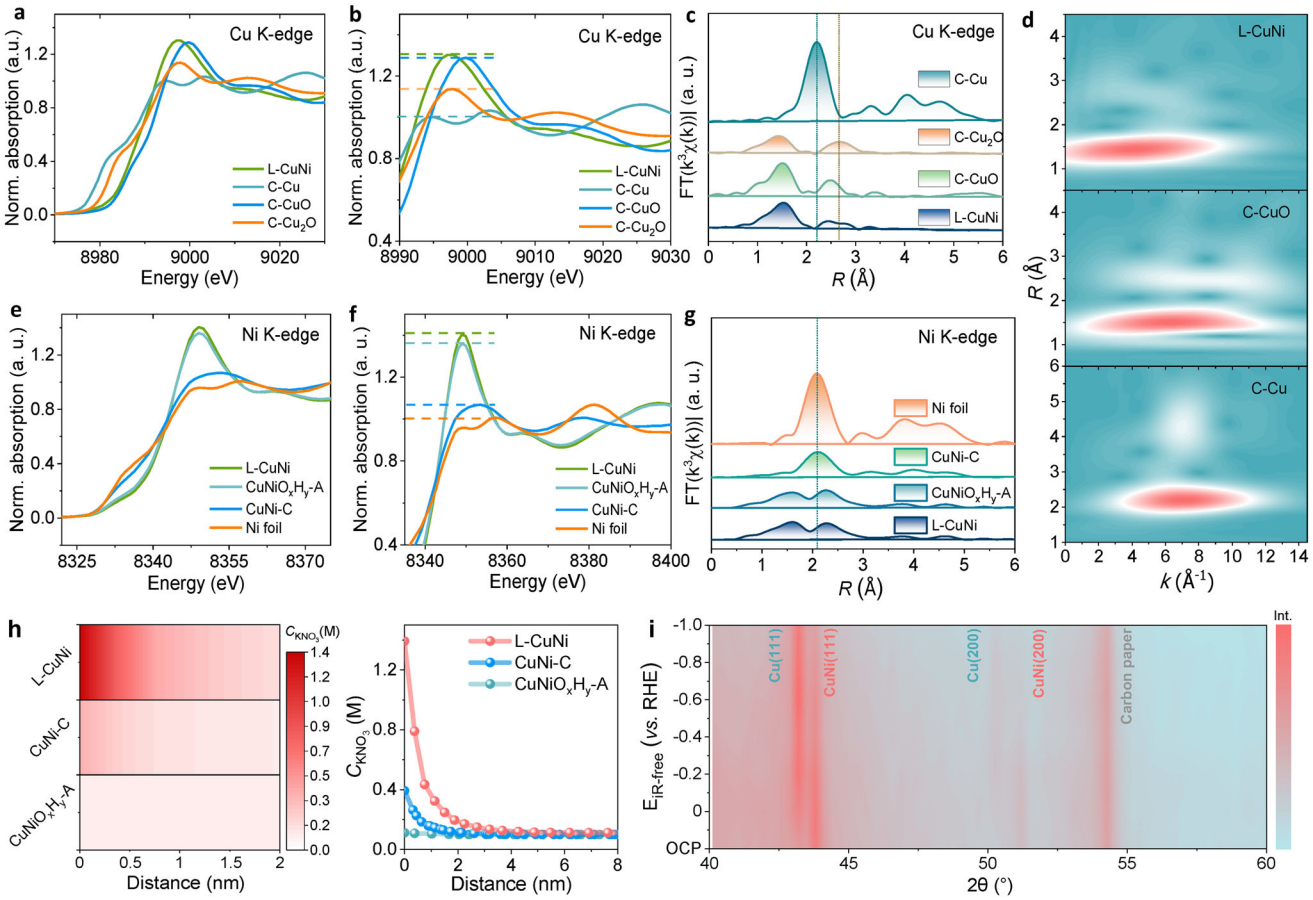
X‐ray techniques for structural characterizations. (a,b) Normalized XANES Cu K‐edge spectra of L‐CuNi, C‐CuO, C‐Cu_2_O, and C‐Cu. (c) Fourier transformed EXAFS and (d) WT–EXAFS Cu K‐edge spectra of L‐CuNi, C‐CuO, C‐Cu_2_O, and C‐Cu. (e,f) Normalized XANES Ni K‐edge spectra of L‐CuNi, CuNiO_x_H_y_‐A, CuNi‐C, and Ni foil. (g) Fourier transformed EXAFS Ni K‐edge spectra of L‐CuNi, CuNiO‐A, CuNi‐C, and Ni foil. (h) The finite element method simulated KNO_3_ distribution and values near the surface of CuNiO_x_H_y_‐A, CuNi‐C, and L‐CuNi through COMSOL multiple physical quantities. (i) X‐ray diffraction during the NITRR process for L‐CuNi.

Then we used X‐ray absorption spectroscopy (XAS) characterizations to obtain information of the electronic and local structure of L‐CuNi. Figure 3a–c show the Cu K‐edge x‐ray absorption near‐edge structure (XANES), and Fourier transform extended x‐ray absorption fine structure (FT‐EXAFS) spectra of L‐CuNi in reference to the commercial C‐Cu_2_O, C‐CuO, and Cu foil. The result shows that the absorption edge of L‐CuNi is obviously shifted to higher energy than that of Cu_2_O and close to that of CuO, which indicates that the average valence of Cu is close to +2 (Figure 3a and Figures S8‐S14 and Tables S1 and S2). In addition, the white line intensities of L‐CuNi are higher than those of C‐CuO, suggesting the lower electron density of surface Cu atoms in L‐CuNi, possibly due to the existence of amorphous CuNiO_x_H_y_ [71]. In the FT‐EXAFS data (Figure 3c), the peak at 2.40 Å of L‐CuNi can be assigned to the representative Cu‐Ni or Cu‐Cu bond [48]; this peak is much weaker than that of CuO, which suggests the loss of long‐range order due to the presence of amorphous CuNiO_x_H_y_. Furthermore, we performed a wavelet transform to visually check the localized coordination environments. As shown in Figure 3d, the wavelet transform maximum intensity for L‐CuNi is ≈4.1 Å^−1^ in the k space for the first coordination shell of Cu‐O, which is smaller than that of C‐CuO. This result suggests the amorphous support structure [72, 73]. Figure 3e–g show the Ni K‐edge XANES and FT‐EXAFS spectra of L‐CuNi, CuNiO_x_H_y_‐A (amorphous CuNiO_x_H_y_), and CuNi‐C (crystalline CuNi, Figures S8 and S9; see Methods for detailed synthesis) in reference to Ni foil. The result shows that, except for the CuNi‐C, the absorption edge of L‐CuNi and CuNiO_x_H_y_‐A are obviously shifted to higher energy than that of Ni foil, which indicates that the average valence of Ni has increased (Figure 3e). Meanwhile, the higher white line intensities of L‐CuNi and CuNiO_x_H_y_‐A compared to CuNi‐C and Ni foil suggest the high oxidation state of Ni (Figure 3f). Meanwhile, the result of XPS also proves that copper and nickel are predominantly in the oxidized state, which is consistent with the results of XANES (Figure S12). In the FT‐EXAFS data (Figure 3g), only CuNi‐C has the peak at 2.10 Å, which can be assigned to the representative Ni‐Ni or Ni‐Cu bond similar to that of Ni foil; the peaks at 1.6 and 2.4 Å can be assigned to Ni‐O and Ni‐Cu/Ni, respectively, which suggests the oxidized Ni [74].

Literature studies suggest that heterostructure interfaces will be beneficial for ion adsorption. Based on the structural characteristics of L‐CuNi, we speculate that an electron concentration gradient will form between the metallic nanoparticles and the metal oxide nanosheets due to their difference in electron structure. Therefore, we investigate the distribution of local ions on the surface of amorphous CuNiO_x_H_y_, nanoparticle CuNi, and L‐CuNi (amorphous CuNiO_x_H_y_/crystalline CuNi) by finite element method (FEM)‐based theoretical simulations performed on COMSOL Multiphysics (Figure 3h and Figure S15) [57, 75, 76]. We selected KNO_3_ as the ions for comparison, the electrolyte for the electrochemical study in this work. We found that the nitrate concentration around the L‐CuNi will rise sharply by 3.5 times and 14 times compared to CuNi nanoparticle or CuNiO_x_H_y_, respectively, which can be ascribed to the different electron densities induced by amorphous/crystalline two‐phase interface [77, 78, 79, 80]. Those results indicate that the local environments arising from the amorphous/crystalline could concentrate the NO_3_^−^ and other species near the catalyst surface, which could promote the ammonia product.

To evaluate the structural robustness of L‐CuNi, we probed the evolution of the crystallography of L‐CuNi under different applied potentials (Figure 3i). Under 0 V, the signal at 42.3° belonging to Cu(111) becomes stronger. This implies the transformation of amorphous CuNiO_x_H_y_ into crystalline Cu/CuNi alloy. From post‐structural analyses in the electrochemical section, it reveals that part of the amorphous structures is converted into crystalline ones, which further increases the crystalline/amorphous interfaces abundance and provides additional active sites for electrochemical reaction.

### 2.3 Nitrate Electroreduction

**FIGURE 4:**
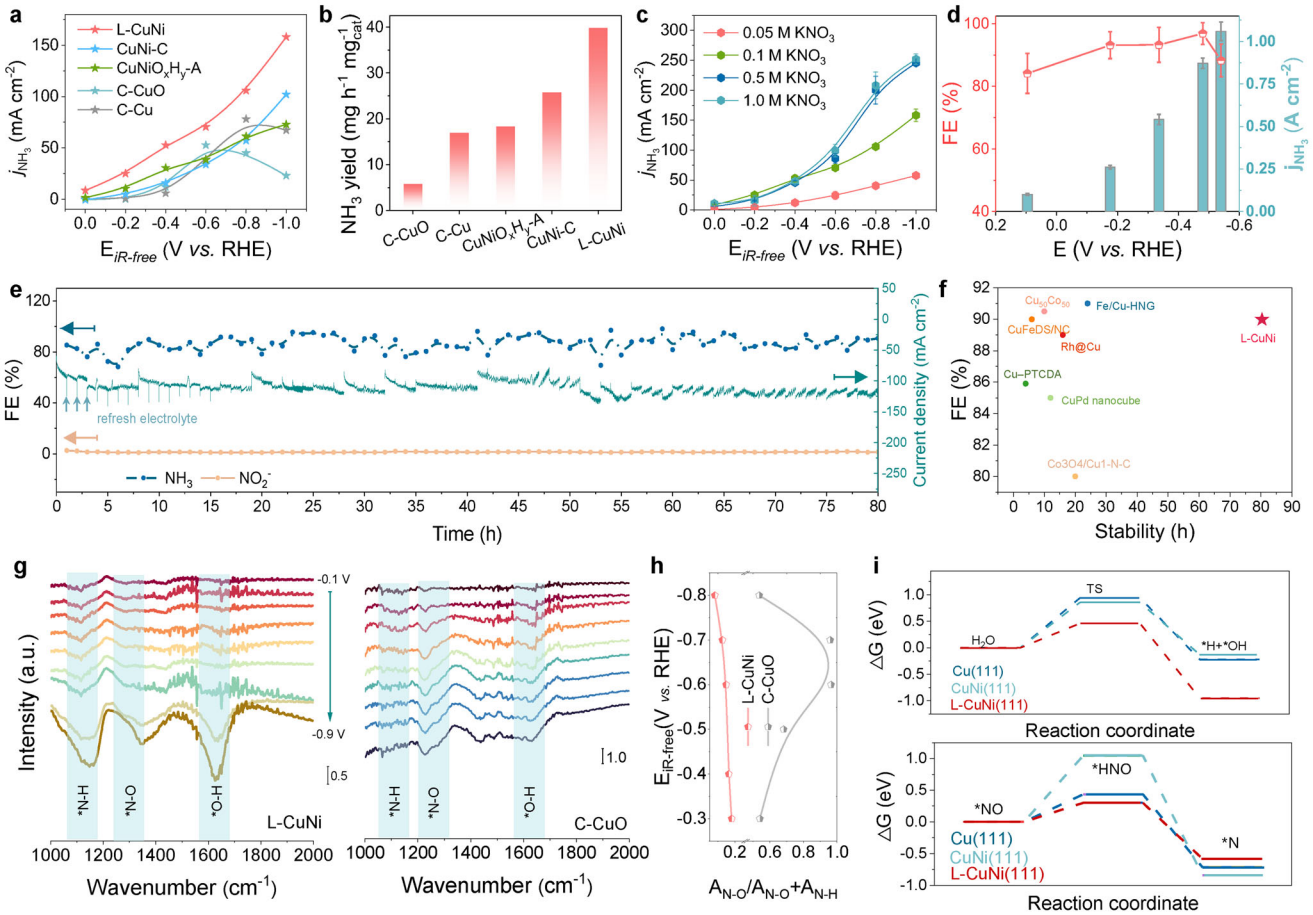
Nitrate electroreduction and catalyst mechanism investigations. (a) Particle density and (b) NH_3_ yield rate of L‐CuNi, CuNi‐C, CuNiO_x_H_y_‐A, C‐CuO, and C‐Cu in 1 m KOH + 0.1 m KNO_3_. (c) Partial NH_3_ current densities of L‐CuNi in 1 m KOH + 0.05 m KNO_3_, 1 m KOH + 0.1 m KNO_3_, 1 m KOH + 0.5 m KNO_3,_ and 1 m KOH + 1 m KNO_3_. (d) FE (lift), j_NH3_ (right) at different potentials of L‐CuNi in 1 m KOH + 1 m KNO_3_ in a flow cell during NITRR. (e) Stability test on L‐CuNi at −0.8 V vs. RHE for 80 h in 1 m KOH + 0.1 m KNO_3_ (electrolyte refreshes every hour). (f) Comparison with that of state‐of‐the‐art catalysts in stability performance. (g) In situ ATR‐FTIR obtained during chronopotentiometry in a potential window from −0.1 to −0.9 V for L‐CuNi (lift) and C‐CuO (right) under NITRR. (h) I_NO_ /I_NH2OH_ +I_NO_ ratio at L‐CuNi and C‐CuO. (i) Water splitting process (top) and free energy of the rate‐determining step process (bottom) over Cu(111), CuNi(111), and L‐CuNi(111).

The electrochemical performance of L‐CuNi for the NITRR was evaluated in a customized H‐cell with different electrolyte compositions under ambient conditions. The performance is also compared to C‐CuO, C‐Cu, CuNiO_x_H_y_‐A, and CuNi‐C. The control samples exhibit much higher crystallinity, as indicated by the strong XRD peak signals (Figures S8‐S14). The concentrations of the products, including nitrite (NO_2_^–^) and NH_3_, were analysed (Figures S16‐S32). The partial current density of NH_3_ of L‐CuNi, CuNi‐C, CuNiO_x_H_y_‐A, C‐CuO, and C‐Cu in 1 m KOH with 0.1 m NO_3_^–^ are shown in Figure 4a. As expected, L‐CuNi always shows the best NH_3_ current density, Faradaic efficiency (FE) under the same potential, indicating its higher activity for the NITRR (Figure 4a). Specifically, from 0 to −1.0 V vs. RHE, the FE of L‐CuNi displays a volcanic shape curve with a maximum of 98.6% at −0.6 V. Meanwhile, the NH_3_ yield rate of L‐CuNi reaches 39.8 mg h^−1^ mg^−1^, which is 1.5, 2.2, 2.4 and 6.9 times higher than that of CuNi‐C, CuNiO_x_H_y_‐A, C‐Cu and C‐CuO (Figure 4b), respectively, suggesting the key role of excellent amorphous/crystalline heterophane structures in the NITRR. We quantified the NH_3_ product concentration as a function of NO_3_^−^ concentrations from 0.05 to 1 m KNO_3_ (Figure 4c and Figures S33‐S37). We achieve a 99 ± 1% FE(NH_3_) at ∼−0.4 V vs. RHE on the L‐CuNi catalyst in 0.05 m KNO_3_ electrolyte. Overall, L‐CuNi shows a high NITRR performance in a wide range of concentrations. Specifically, the highest FE(NH_3_) are 94.6 ± 13.4%, 98.6 ± 5.3%, 99.1 ± 11.5%, and 94.5 ± 10.4% in 0.05, 0.1, 0.5, and 1 m NO_3_^−^, respectively. The corresponding NH_3_ yield rates generally increase with nitrate concentrations, reaching the highest of 65.2 mg h^−1^ mg^−1^ in 1 m KNO_3_.

We further demonstrate the industrial‐relevant NITRR performance by using a flow cell to improve the cascade reactions. Impressively, L‐CuNi achieves a high *j*_NH3_ of 1.08 A cm^−2^ with a splendid NH_3_ yield rate of ∼92.18 mg h^−1^ cm^−2^ (Figure 4d and Figure S38). The current generated by the bare CC in the whole potential window is negligible, excluding the influence of the substrate (Figure S30).

We also characterized the stability of L‐CuNi at −0.8 V vs. RHE (Figure 4e). While the FE is stable during the 80‐h test, the *j* increases from 75 to ∼120 mA cm^−2^. Post‐structural analyses show that L‐CuNi still retains the amorphous/crystalline interfaces, but additional nanocrystals are produced during the electrocatalytic process (Figures S39‐S43). The stability primarily stems from the unique amorphous support that resists structural disruptions throughout electrocatalysis [81]. Compared to most reported catalysts, our catalyst demonstrates excellent performance in both ammonia selectivity and stability (Figure 4f and Figure S44, and Table S3).

To probe the dynamic evolution of surface adsorbed intermediates and products during NTIRR and to elucidate the mechanism of boosted NH_3_ selectivity and yield, we performed in situ attenuated total reflection Fourier transformed infrared spectroscopy (ATR‐FTIR) and the online differential electrochemical mass spectrometry (DEMS). In the in situ ATR‐FTIR, with the potential increasing from 0 to −1.0 V, the absorption bands at 1354 and 1236 cm^−1^ emerge, which are attributed to N‐O antisymmetric stretching vibration of NO_3_^−^ and NO_2_^−^, respectively; another intermediate observed around 1110 cm^−1^ is ascribed to N‐H stretching vibration of *NH_2_, which is a key intermediate for NH_3_ formation [39, 82]. The band at 1650 cm^−1^ from the O‐H peak of L‐CuNi increases much faster than that of C‐CuO, indicating the excellent H_ads_ generation ability of L‐CuNi (Figure 4g) [55]. In addition, with the negative shift of the working potential, the ratio of area of N‐O relative to the sum area of N‐O and N‐H on L‐CuNi drops more sharply than that of C‐CuO, especially in high potential, which suggests that L‐CuNi accelerates the hydrodeoxygenation of NO_2_^−^ enhanced, thus improving the formation of the final product NH_3_ (Figure 4h). Meanwhile, DEMS test shows the following m/z signals: NH_3_(17), NO (30), and NH_2_OH (33) on L‐CuNi during NITRR (Figures S45 and S46). These signals align periodically with the applied potentials. The signals of NO and NH_2_OH are two orders of magnitude lower than NH_3_, implying their unfavorable production. The in situ spectroscopic data are consistent with the observed electrochemical performance.

To further verify the mechanism, we construct single Cu, CuNi, and L‐CuNi as model catalysts. Cu (111), crystalline CuNi (111), and CuNi (111) with amorphous/crystalline interfaces are selected based on the results of the STEM (Figures S47‐S57). The Gibbs free energy change (ΔG), as well as the corresponding adsorption configurations from NO_3_^−^ to NH_3_ on the three surfaces, are summarized in Figure 4i and Figure S50. Figure 4i (top) displays the calculated energy profiles of the H_2_O dissociation process involving the initial, transition, and final states. The energy barrier of H_2_O dissociation for L‐CuNi (0.46 eV) is lower than that of CuNi (0.86 eV) and Cu (0.94 eV), indicating that the introduction of amorphous structure is beneficial for the dissociation of H_2_O on L‐CuNi to facilitate the formation of *H for N‐intermediate species hydrogenation [83]. The hydrogenation conversion from NO* to HNO* is identified as the RDS for the three surfaces. L‐CuNi (111) has the smallest ΔG with an uphill of 0.3 eV, while the RDS for Cu (111) and CuNi (111) show a much larger ΔG of 0.43 eV and 1.05 eV, respectively. This result highlights the critical role of amorphous/crystalline interfaces in facilitating the NITRR (Figure 4i bottom).

**FIGURE 5:**
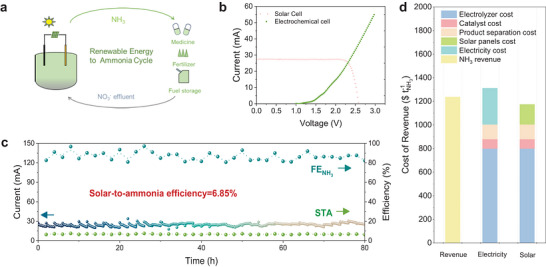
Solar to ammonia coupled systems. (a) Schematic diagram of Renewable Energy to Ammonia. (b) I‐V curves of the solar cell and the two‐electrode electrolysis cell for L‐CuNi. (c) Current density, FE(NH_3_), and solar‐to‐ammonia efficiency vs. time for L‐CuNi (electrolyte refreshes every 2 h). (d) Technoeconomic analysis of different pathways for ammonia production.

We finally demonstrate the conversion of nitrate into ammonia using solar energy, an attractive strategy for storing renewable energy (Figure 5a and Figures S58‐S60). This can be achieved by coupling photovoltaics (PV) to electrochemical cells (EC). Since practical nitrate sources such as industrial wastewater have low nitrate concentrations, we chose 50 mm KNO_3_ as the electrolyte. Because the full EC includes the other half‐reaction of the oxygen evolution reaction, which typically demands a cell voltage of >1.4 V, here we connect two commercial GaInP/GaInAs/Ge triple‐junction solar cells with an area of 1 cm × 1 cm in parallel as the power source. The current‐voltage (I‐V) curve is obtained under simulated AM 1.5G illumination with the intensity of 100 mW cm^−2^ (Figure 5b). The two I‐V curves cross at the point (2.31 V, 25.29 mA), which is close to the maximum power point of the PV module. During the 80 h stability test powered by a solar cell, an FE(NH_3_) of over 80% is obtained. The solar‐to‐ammonia efficiency is as high as 6.85%. There is no appreciable decay in activity and selectivity of L‐CuNi during the 80 h stability test (Figure 5c). To evaluate the practical application prospects of the solar‐to‐ammonia system, we carried out a techno‐economic analysis in reference to the market price of ammonia. It is estimated that the ammonia price is roughly 1174.7$ per ton, lower than the market price of 1237$ per ton. Considering the potential environmental benefits, the PV‐EC NITRR system is undoubtedly an attractive and competitive route for wastewater treatment (Figure 5d and Note S1, and Table S4).

### 2.4 Extended Synthesis

**FIGURE 6:**
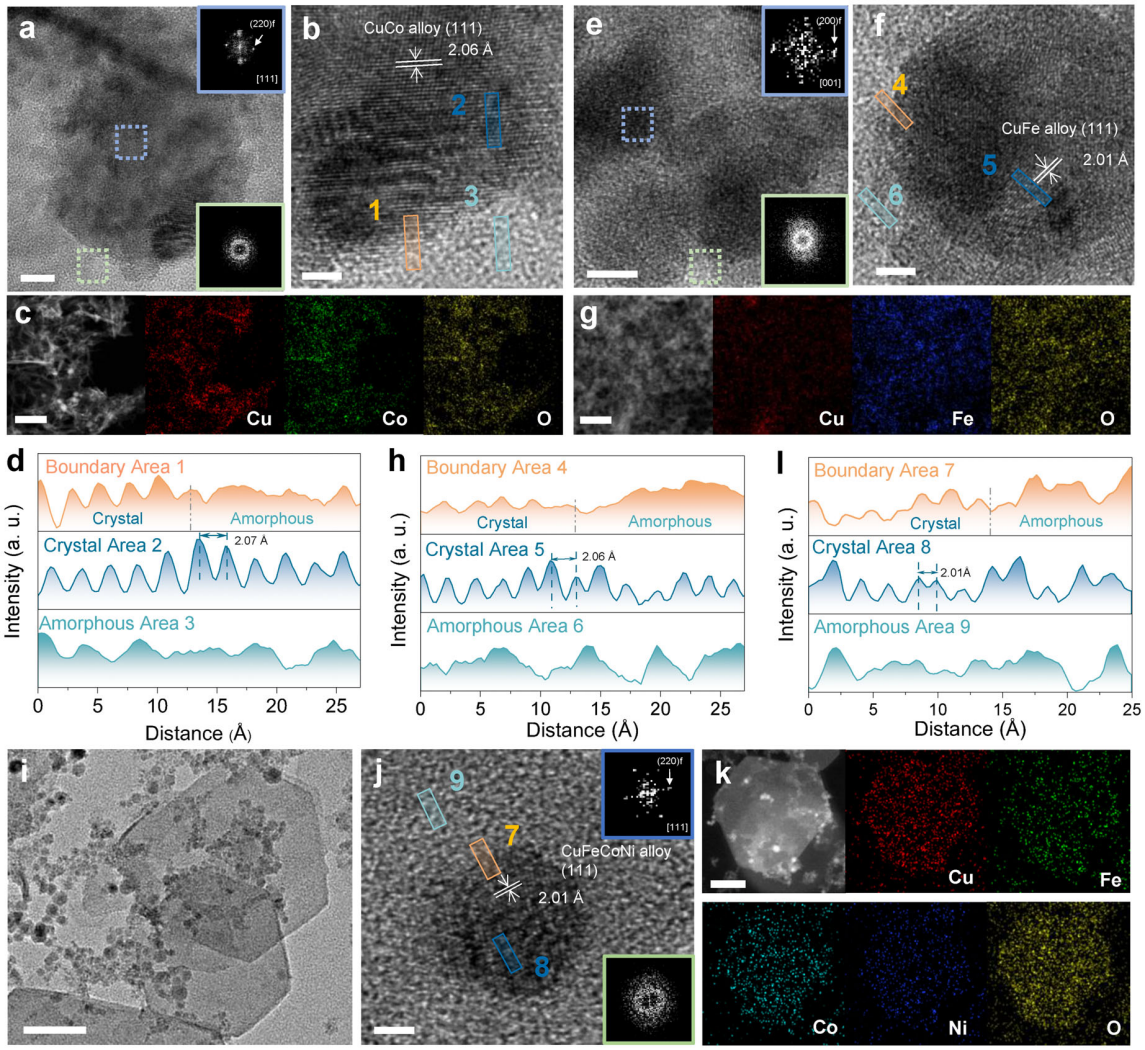
Morphology characterization of L‐CuCo, L‐CuFe, and L‐CuFeCoNi. HR‐TEM image and FFT patterns of the crystalline zone and amorphous zone (a), HR‐TEM images (b), and EDX mapping (c) of L‐CuCo. (d) The line intensity profile acquired along the areas in (b). HR‐TEM image and FFT patterns of the crystalline zone and amorphous zone (e), HR‐TEM images (f), and EDX mapping (g) of L‐CuFe. (h) The line intensity profile acquired along the areas in (f). TEM image (i), HR‐TEM images and FFT patterns of the crystalline zone and amorphous zone (j), and EDX mapping (k) of L‐CuFeCoNi. (l) The line intensity profile acquired along the areas in (j). Scale bar: 5 nm in (a and e), 2 nm in (b, f, and j), and 50 nm in (c, g, i, and k).

We further characterized the laser‐synthesized L‐CuFe and L‐CuCo structures using electron microscopy (Figure 6 and Figures S61 and S62). Both L‐CuFe and L‐CuCo exhibit structural features similar to those observed in L‐CuNi. Figure 6a shows that L‐CuCo consists of ultra‐thin nanosheets decorated with numerous nanoparticles. The FFT patterns (Figure 6a,b) reveal diffraction rings corresponding to crystalline CuCo planes, confirming the crystalline nature of the nanoparticles, while the absence of distinct rings in the flake‐like regions indicates their amorphous character. These results confirm that the ultrathin nanosheets are composed of amorphous CuCoO_x_H_y_, whereas the nanoparticles are crystalline CuCo. EDX elemental mapping confirms the homogeneous distribution of Cu, Co, and O throughout the sample (Figure 6c). HRTEM images clearly distinguish the atomic‐scale features between the amorphous nanosheets and crystalline nanoparticles (Figure 6d). An intensity profile taken across the amorphous‐crystalline interface (Figure 6d top) illustrates the transition between the two phases, reflecting expanded interatomic spacing in the crystalline CuCo region. The crystalline domains exhibit relatively uniform interatomic distances and intensity variations, as clearly observed in the surface intensity profile. In contrast, the CuCoO_x_H_y_ nanosheets show a completely disordered structure, with random fluctuations in both intensity and atomic spacing (Figure 6d bottom).

A similar structural configuration is evident in the L‐CuFe sample (Figure 6e,f). The FFT patterns acquired from the nanoparticle and nanosheet regions show distinct features corresponding to crystalline and amorphous phases, respectively. EDX mapping reveals a homogeneous distribution of Cu, Fe, and O elements (Figure 6g). Atomic‐scale intensity profiles (Figure 6h) further support these observations: the crystalline regions display regular interatomic distances, while the amorphous nanosheets exhibit completely disordered surfaces.

Building upon the successful synthesis of the binary systems, we further extended the composition space to a medium entropy system. As shown in Figure 6i, L‐CuFeCoNi particles are uniformly dispersed on the nanosheets. HRTEM image reveals distinct structural differences between the two components: the particles exhibit well‐defined lattice fringes, indicative of their crystalline nature, while the supporting nanosheets show diffuse diffraction halos, characteristic of an amorphous matrix (Figure 6j, Figure S63–S65). This morphological and structural characterization is corroborated by elemental analysis. EDX mapping confirms the homogeneous spatial distribution of all four metallic elements (Cu, Fe, Co, and Ni) throughout the architecture (Figure 6k). Atomic‐scale intensity profiles (Figure 6l) reveal that the crystalline regions display periodic intensity variations and consistent interatomic distances, affirming long‐range order. In stark contrast, the amorphous nanosheets exhibit a completely random intensity distribution with no discernible periodicity, supporting their non‐crystalline structure. Collectively, this laser‐assisted technique proves to be an effective and generalizable strategy for producing crystalline particles anchored on amorphous nanosheets. Its efficacy is demonstrated across a range of metallic systems, from binary to quaternary compositions.

## 3 Conclusions

In summary, our study presents a laser‐based strategy for synthesizing highly active catalysts exemplified by the amorphous metal oxide‐supported crystalline metals (L‐CuNi, L‐CuFe, L‐CuCo, and L‐CuFeCoNi). This method enables the production of a range of nanomaterials consisting of crystalline alloys anchored on amorphous oxide supports with room‐temperature operation, one‐step synthesis, and water‐based processing. Electron microscopy and spectroscopic analyses reveal the mechanistic steps of the synthesis, establishing a foundation for extending this approach to other material systems. Through comprehensive in situ characterization, finite element simulations, and density functional theory calculations, we gain deep insights into the electronic interactions between the amorphous oxide support and crystalline metal, the electronic redistribution induced by this unique structure, and the dynamic reconstruction of active sites during reaction—all critical factors for enhancing ammonia production rate and catalytic stability. Our laser‐based fabrication strategy is expected to inspire the design of next‐generation catalysts for sustainable chemical conversion.

## 4 Experimental Section

### 4.1 Material Synthesis

**Preparation of L‐CuNi and CuNiO_x_H_y_‐A**. To synthesize L‐CuNi, a pulsed Nd:YAG laser (Nimma‐600, Beamtech) was utilized to ablate a CuNi target submerged in deionized water (Movie S1). The laser parameters were as follows: wavelength of 1,064 nm, and pulse frequency of 15 Hz. The CuNi material used was of 99.99% purity and was shaped into a rectangular target measuring 20*90 mm in length and 5 mm in thickness. Prior to laser ablation, the target was placed in a 50 mL beaker filled with deionized water, with the water level positioned 10 mm higher than the target surface. The laser ablation process lasted for 60 min, resulting in the formation of a colloid solution containing CuNi. This solution was then subjected to centrifugation at 13,320 × g for 15 min, repeated three times, to enrich the CuNi content. The CuNiO_x_H_y_‐A were prepared by following the same procedure as for the L‐CuNi, except that the CuNi target was placed in a 100 mL beaker, and after laser, the solution was then subjected to centrifugation at 6,320 × g for 15 min.

**Preparation of CuNi‐C**. First added 5 mg Cu(acac)_2_, 5 mg Ni(acac)_2_, 20 mL oleylamine, and 4 mL 1,2 Butanediol into a 50 mL round‐bottom flask and then heated the mixture at 180° for 5 h. After cooling, it was divided into two tubes and centrifuged 3 times at 13,320 × g for 15 min.

Preparation of L‐CuCo, L‐CuFe, and L‐CuFeCoNi. The L‐CuCo, L‐CuFe, and L‐CuFeCoNi were prepared by following the same procedure as for the L‐CuNi, except that the target changed to Cu_50_Co_50_, Cu_50_Fe_50,_ and Cu_25_Fe_25_Co_25_Ni_25_, respectively.

### 4.2 Characterizations

The high‐angle annular dark‐field scanning transmission electron microscopy (HAADF‐STEM) technique, in combination with energy‐dispersive X‐ray spectroscopy (EDX), was utilized to capture STEM and mapping images at an accelerating voltage of 200 kV. X‐ray diffraction (XRD) patterns of the samples were obtained using a Bruker D2 instrument with a Cu Kα source. The scan step was set at 10° min^−1^, covering a range between 10° and 80°. X‐ray photoelectron spectroscopy (XPS) data were collected using a Thermo ESCALAB 250Xi spectrometer equipped with a monochromatic AlK radiation source (1486.6 eV) and a pass energy of 20.0 eV. The data were calibrated using the C 1s peak at 284.8 eV. X‐ray absorption fine structure (XAFS) measurements were conducted in transmission mode at the X‐ray absorption fine structure for catalysis at the beamline of the Singapore Synchrotron Light Source. The synchrotron operated at 700 MeV with a beam current of 200 mA. Data processing was carried out using the Athena and Artemis software packages. In situ electrochemical Fourier‐transform infrared (FTIR) measurements were performed using a Thermo iS50 instrument. Online differential electrochemical mass spectrometry (DEMS) analysis was employed to analyze the intermediates and products of catalysts during NITRR in a flow cell. EELS spectra were acquired using an aberration‐corrected transmission electron microscope (JEOL ARM 200F) equipped with a cold field emission gun, a condenser lens aberration corrector system, an energy filter system (Gatan 1065), and a CMOS camera (Gatan 1095). The acceleration voltage was set to 200 kV. The STEM probe current was approximately 17 pA, with a probe size of 0.133 nm. The STEM convergence angle was 20.6 mrad, and the collection angle for EELS was 54–220 mrad. The energy resolution, determined by the full width at half maximum (FWHM) of the zero‐loss peak, was better than 0.5 eV. All EELS spectra were processed using DigitalMicrograph software (Gatan Inc.). Background subtraction was performed using a power‐law model fitted to a pre‐edge region of approximately 30–50 eV before the L_3_ edge. The L_3_ and L_2_ white‐line intensities for Cu and Ni were obtained by integrating the background‐subtracted spectra over energy windows of 5–10 eV centered at each peak. The L_3_/L_2_ intensity ratios were then calculated to evaluate the oxidation states, following standard procedures established in the literature.

### 4.3 Electrochemical Measurements

Preparation of the working electrode. The obtained L‐CuNi powder was redispersed in 1000 µL of ethanol, followed by the addition of 20 µL of Nafion solution (5 wt.%) and subsequent sonication for 30 min to make a homogeneous catalyst ink. Then, 60 µL of catalyst ink was dropped onto the carbon paper. The total area of carbon paper is 0.5 cm × 2 cm, and the catalyst ink is dripped on two sides of 0.5 cm ×1 cm at one end of the carbon paper to ensure that the entire area entering the electrolyte is 1 cm^2^. After the ink was dry, the prepared working electrode was kept in a vacuum atmosphere to avoid oxidation before the electrocatalytic test.

**Electrochemical reduction of potassium nitrate in an H‐cell**. All NITRR experiments were conducted utilizing a three‐electrode setup in a dual‐compartment H‐cell, which was divided by an ion‐exchange membrane (Nafion 117) and linked to a CHI 650 electrochemical workstation (Chenhua, Shanghai). The prepared L‐CuNi was loaded onto 0.5 cm^2^ carbon paper with 0.6 mg_catalyst_ cm^−2^, Hg/HgO, and a platinum plate, which served as the working electrode, reference electrode, and counter electrode, respectively. A 15 mL mixture of KOH/KNO_3_ solution (in varying configurations) was employed as both the cathode and anode electrolyte. All potentials were measured against the reversible hydrogen electrode (RHE) using the formula ERHE = E_Hg/HgO_ +0.0591*pH + 0.098. Cyclic voltammetry (CV) and LSV were conducted at scan speeds of 10 and 5 mV s^−1^, respectively. Potentiostatic tests were performed at various potentials for 3600 s at a stirring speed of 800 rpm. After 1 h of potentiostatic tests in different potentials, we collected the liquid product to measure the amount. Specifically, the liquid product was transferred to the sample collection tube by a pipette, and then the electrolytic cell was rinsed three times with the corresponding electrolyte, after that, fresh electrolyte was added to the electrolytic cell for the next potential test. It is carried out at room temperature and pressure. The potential range for calculating the NH_3_ FEs and yield rates ranged from 0 V to −1.0 V vs. RHE, with intervals of −0.1 V. Isotopic labeling trials were conducted using the same techniques at −0.8 V vs. RHE, except that the nitrogen source was replaced with 99% ^15^NO_3_^−^. Unless otherwise stated, all tests were conducted in an environmental chamber at room temperature without iR‐compensation.

**Electrochemical reduction of potassium nitrate in a flow‐cell**. The flow cell was three chambers with the anolyte and catholyte circulating by a peristaltic pump operating with 10 mL min^−1^. The Ar gas flow rate was controlled using a mass flow controller and set to 10 sccm. The catalyst ink was dropping in the 1 cm * 2 cm carbon paper with 1 mg_catalyst_ cm^−2^. 1 m KOH and 1 m KNO_3_ solution was used as the anolyte and catholyte.

**Product detection**. The gas and liquid products under different potentials during NITRR through UV–vis spectrophotometry and nuclear magnetic resonance (NMR).

**Ammonia detection**.^1^H nuclear magnetic resonance (^1^H NMR) was recorded on an AVANCE III HD 300 system to detect ammonia. The pH value of the final electrolyte was adjusted to be weakly acidic with 2 M HCl. Maleic acid (C_4_H_4_O_4_, 50 ppm) was employed as the external standard to calibrate the standard curve of NH_4_^+^ using the peak area ratio between NH_4_^+^ and maleic acid. The isotope labeling experiments were also measured through the same process.

**Nitrite detection**. The concentration of nitrite was determined using UV–vis spectrophotometry following the standard procedure. Initially, a color reagent was prepared using a blend of p‐aminobenzene sulfonamide (4 g), N‐(1‐naphthyl) ethylenediamine dihydrochloride (0.2 g), deionized water (50 mL), and phosphoric acid (10 mL, *ρ* = 1.685 g ml^−1^). The electrolyte sample was then procured and diluted to fall within the detection range. Subsequently, 40 µl of the color reagent was combined with a 2.0 mL sample solution, mixed well, and left to rest for 20 min under normal conditions. The absorption intensity at a wavelength of 540 nm was subsequently measured using a UV–vis spectrophotometer (UV‐2600). A concentration‐absorbance curve was established using a series of standard potassium nitrite solutions that were linearly fitted and prepared in advance. Finally, the concentrations of the nitrite product were computed based on the tested absorbance and the established standard curve.

The Faradaic efficiency (FE) and yield were calculated according to the following equations:

(1) $$FE_{liquid}=\frac{Q_{liquid}}{Q_{total}}\times 100\%=\frac{n_{liquid}\times N\times F}{j\times t}\times 100\%$$

(2) $$Yield_{NH_{3}}=\frac{n_{NH_{3}}\times M_{NH_{3}}}{m_{Catalyst}\times t}$$

*n* is the amount of product (mol), *N* is the number of electron transfer to form a molecule of product; *F* = 95200 C mol^−1^ is Faraday constant, *T* = 298 K is the temperature (K) and *R* is the molar gas constant = 8.31 J (mol K) ^−1^, *j* is the total current, *t* is the electrolysis time (s), *M* is the Molar mass of product (g mol^−1^), *m* is the quality of catalyst (mg).

**In situ FTIR spectroscopy**. In situ FTIR spectra were acquired in a three‐electrode cell with a Thermo Scientific Nicolet iS50 equipped. Ag/AgCl and Pt wire were used as the reference electrode and counter electrode, respectively. 0.1 M KNO_3_ was taken as the electrolyte. Each spectrum was recorded by 32 scans at an 8 cm^−1^ spectral resolution.

**Online DEMS**. The flow cell was used during the DEMS measurements. Carbon paper coated with L‐CuNi electrocatalysts, Ag/AgCl, and a platinum plate were used as the working electrode, reference electrode, and counter electrode, respectively. LSV technology was employed from 0 to −1.0 V at a scan rate of 10 mV s^−1^ until the baseline kept steady. Then, the corresponding mass signals appeared. After the electrochemical test was over and the mass signal returned to baseline, the next cycle was started using the same test conditions to avoid accidental errors during DEMS measurements. Six cycles were performed for the DEMS experiment.

**Practical applications of solar‐to‐ammonia combined system**. The configuration of the NITRR H‐cell was powered by two commercial GaInP/GaInAs/Ge tripe‐junction solar cells, which were parallel‐connected. The PV‐EC system was operated under 1 sun illumination (AM1.5G) using a solar simulator light source (Zolix, GLORIA‐X500A), and the light intensity was calibrated with a Si reference (Zolix). The full cell voltage and current of PV‐EC system were measured by a source meter (Keithley, 2612B). The solar‐to‐ammonia conversion efficiency (STA) was calculated by the following equation:

(3) $$\eta_{STA}=\frac{P_{out}}{P_{in}}=\frac{I_{OP}\times E_{0}\times FE}{P_{light}\times A}$$

*I_OP_* is the operating current of NO_3_^–^‐to‐NH_3_, *E_0_* is the thermodynamic potential of NO_3_^–^‐to‐NH_3_ (0.69 V vs. RHE), *FE* is the selectivity of NO_3_^–^‐to‐NH_3_, *P_light_* is the input power of the system (100 mW cm^−2^), *A* was the area of the solar cell (2 cm^2^).

### 4.4 Computational Methods

Spin‐polarized density functional theory (DFT) calculations were performed with the Vienna ab initio simulation package (VASP) [84], using projector augmented wave (PAW) pseudopotential for the core electrons, a cutoff energy of 450 eV for the valence electrons, and the generalized gradient approximation (GGA) in the form of Perdew−Burke−Ernzerhof (PBE) for the exchange correlation potentials [85, 86]. The atoms were relaxed fully until the energy convergence reached 0.00001 eV and the force acting on each atom was less than 0.02 eV/Å. Van der Waals (vdW) interaction was considered at the DFT‐D2 level as proposed by Grimme (IVDW = 12) [87, 88, 89]. Initial structures of Cu (mp‐30) were obtained from the Materials Project database [90]. The CuNi alloy was obtained by replacing one Cu atom of mp‐30 with Ni atom. The CuNiO_2_‐supported CuNi model was built by stacking the CuNi(111) surface on the CuNiO_2_ (mp‐1178369) (111) surface. To make the CuNiO_2_‐supported CuNi model amorphous, the model was subjected to ab initio molecular dynamics (AIMD) calculations. AIMD simulations were run for 20 ps as equilibration with time steps of 2 fs, performing a constant temperature of 300 K in the Nosé‐Hoover canonical ensemble. The NO_3_^−^ reduction reactions were modeled based on the computational hydrogen electrode model (CHE) [91], where the free energy of a proton and electron pair is half of the free energy of a free hydrogen gas molecule. The Gibbs free energy change for each reaction step is calculated as,

(4) $$\Delta G=\sum G\left(products\right)-\sum G\left(reac\tan ts\right)$$

where *G*(*i*) is the Gibbs free energy of species *i*. Gibbs free energy of each species was calculated as *G*  = *E* + *ZPE* − *TS*, where E is the total energy obtained from DFT calculations; ZPE is the zero‐point energy, and S is the entropy. Temperature *T* was set to be 300 K.

### 4.5 The Finite Element Method (FEM) Based Theoretical Simulations

The FEM‐based theoretical simulations were performed on COMSOL Multiphysics with the same procedure as in the previous references [57, 76, 92]. To simplify the theoretical model, the 2D models were constructed, which represent the surface of the materials. For simplification of the calculations, the size of the CuNi nanospheres was set to 10 nm with a spacing of 1 nm. The model utilized three parallel CuNi nanospheres to simulate the concentration of NO_3_^–^ around them (electrolyte: 1 m KNO_3_). The CuNi/CuNiO_2_ was simplified as a nanosheet (500 nm), with CuNi nanospheres embedded on the surface. The NO_3_^−^ion diffusion process can be modeled and calculated by the following Poisson–Nernst–Planck equations,

(5) $$\nabla \cdot \left(D\nabla c_{i}+\frac{Dz_{i}e}{k_{B}T}c_{i}\nabla V\right)=0$$

where, *c, D, z, e, k_b_, and T* are the ion concentrations, diffusion coefficients, ion valences, the elementary charge, Boltzmann constant, and the absolute temperature, respectively. To ensure the high accuracy of the simulation result, the densest conventional triangular meshes were used for all simulations on the surface. The mesh solver was used with a relative tolerance of 0.001.

## Author Contributions

R.Y. conceived and designed the research. R.Y., B.T., Z.G., and T.Y. supervised the research. W.G. carried out most of the experiments with assistance from S.Z. and Y.M. J.Z. provided the target material, and S.Z. performed the calculations. Y.M. and Z.G. performed the in situ Fourier transform infrared studies. S.X. and S.Z. performed the X‐ray absorption spectroscopy experiment. R.Y. and W.G. analysed the data and wrote the manuscript with input from the other authors.

## Conflicts of Interest

The authors declare no conflicts of interest.

## Supporting information




**Supporting File 1**: adma73764‐sup‐0001‐SuppMat.pdf

**Supporting File 2**: adma73764‐sup‐0002‐MovieS1.mp4

## Data Availability

The data that support the findings of this study are available in the supplementary material of this article.
