# Supplementary material for: Transient Laser‐Shocked Synthesis of Amorphous Layer‐Supported Metal Nanocrystals for Efficient Nitrate Reduction
Source: Adv Mater. 2026 Jun 18;38(41):e73764. doi: 10.1002/adma.73764 (PMC13393962; doi:10.1002/adma.73764)
Supplement: Supplementary file 1 — Supporting File 1: adma73764‐sup‐0001‐SuppMat.pdf [file ADMA-38-e73764-s001.pdf]

Supporting Information

Transient Laser-Shocked Synthesis of Amorphous Layer-Supported Metal Nanocrystals for Efficient Nitrate Reduction

Weihua Guo[1,2,†], Jixun Zhang[3,†], Siwei Zhang[4,†], Yangbo Ma[5,†], Yun Song[1,2,], Jianjun Su[1,2,], Zihao Li[1,2], Yinger Xin[1,2], Qiang Zhang[1,2], Mingming He[1,2], Ruixuan Wang[1,2], Rui Xue[1,2], Ge Ye[1,2] Shibo Xi[6], Shenlong Zhao[7], Tao Yang[3,], Zhengxiao Guo[5,*], Ben Zhong Tang[4,6,*], Ruquan Ye[1,2,*]*

*Corresponding author.

E-mail: ruquanye@cityu.edu.hk; tangbenz@cuhk.edu.cn; zxguo@hku.hk; taoyang6@cityu.edu.hk.

This PDF file includes:

Figures S1-S65
Tables S1-S4
Note S1
SI References

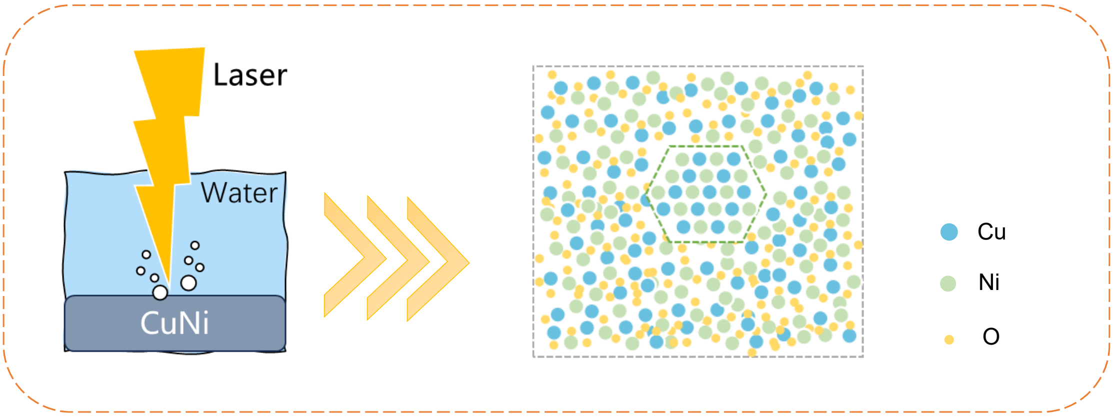

Figure S1. Schematic illustration for the synthesis of L-CuNi by laser.

L-CuNi was prepared facilely by laser irradiation of a bulk CuNi target immersed in deionized water, as shown in Figure S1. The transient heating ablates interfacial CuNi into a vapor state, which is subsequently quenched by cool water into a solid state. As such, the bulk CuNi is gradually transformed into dispersive L-CuNi.

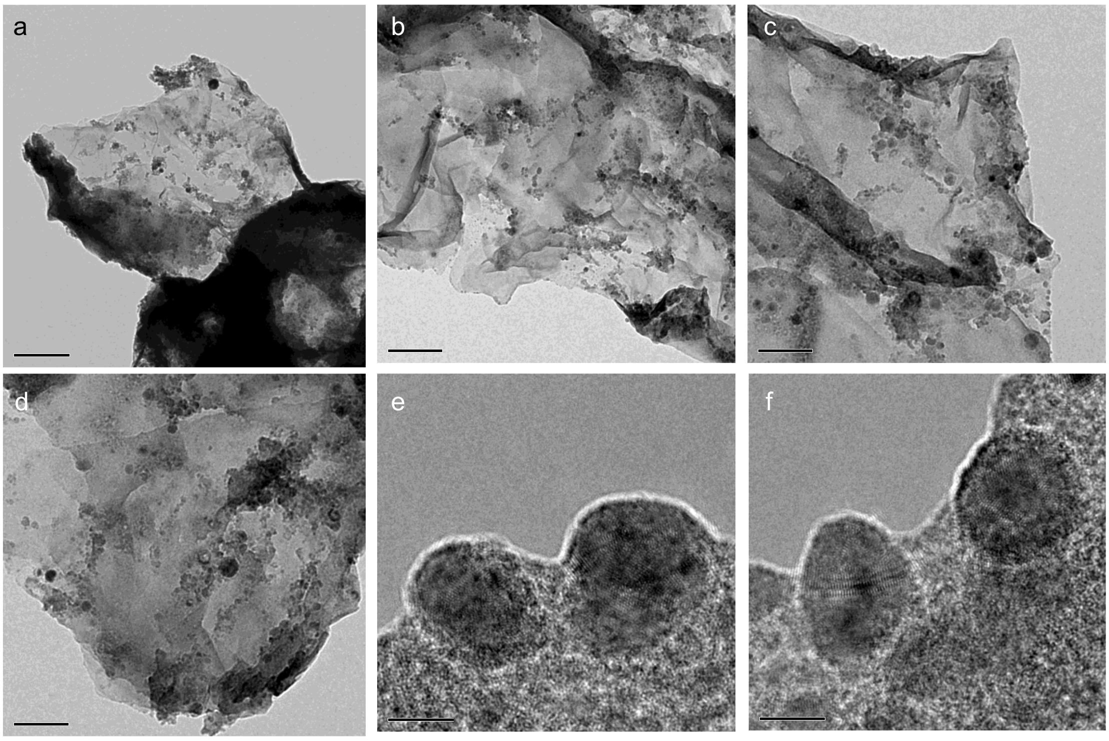

Figure S2. Structural characterization of L-CuNi. (a-d) TEM images and (e and f) HR-TEM images of L-CuNi.

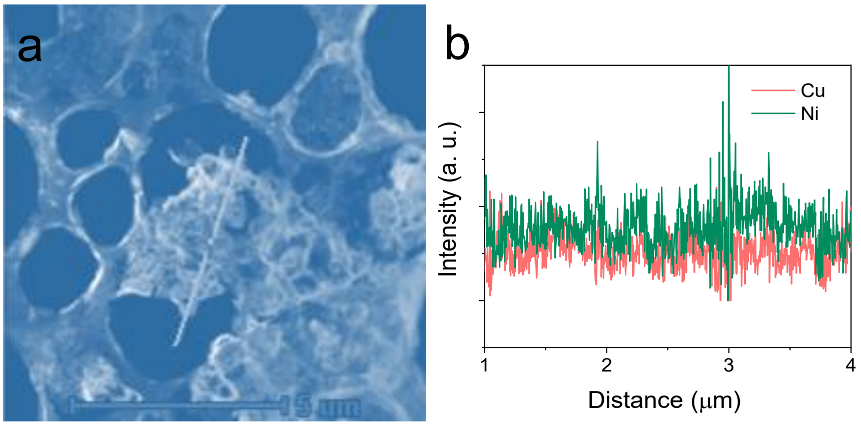

Figure S3. Structural characterization of L-CuNi. (a) Line scan and (b) the corresponding copper-nickel signal of L-CuNi.

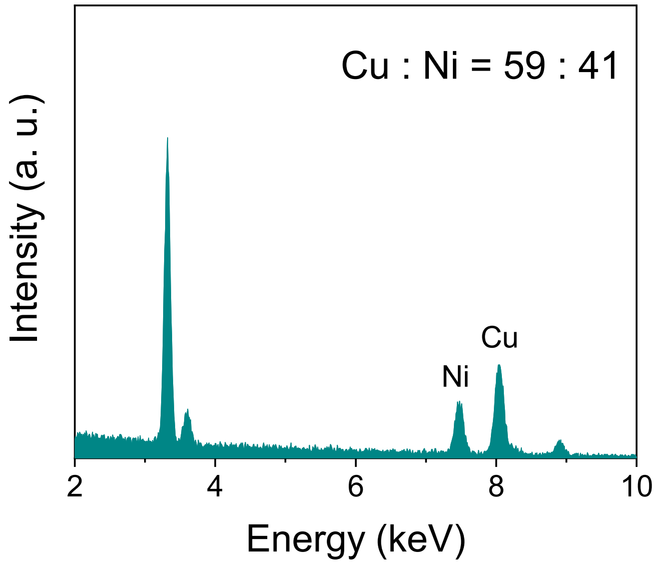

Figure S4. Composition characterization of L-CuNi. Typical EDS spectra of L-CuNi.

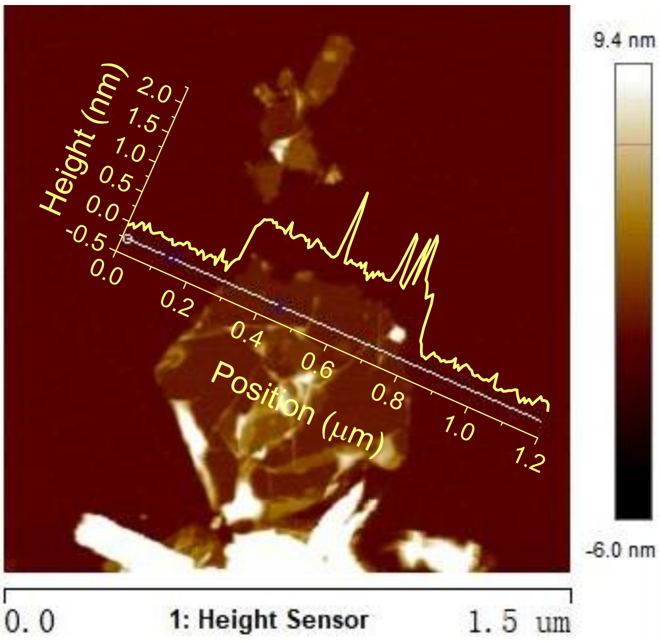

Figure S5. AFM image and corresponding height measurements of L-CuNi. The thickness is below 1 nm, which is almost single atom layer which may be due to the amorphous structure of nanosheet.

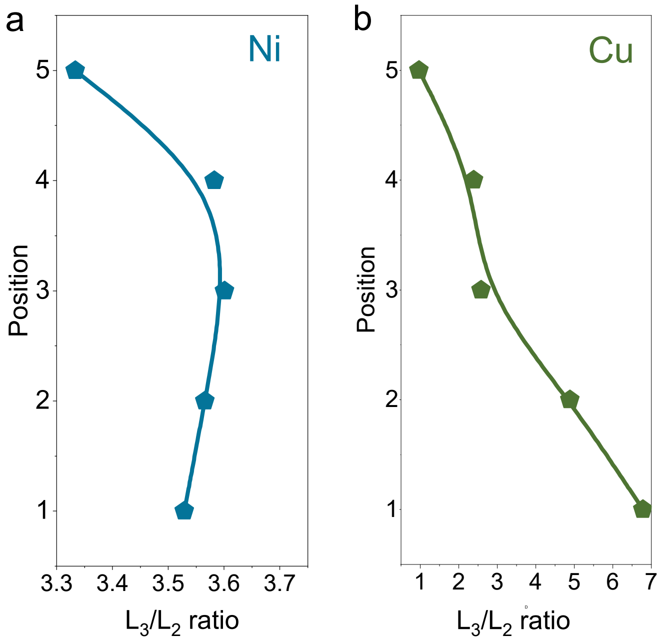

Figure S6. (a) Ni-$L_{2,3}$ and (b) Cu-$L_{2,3}$ white-line ratios extracted from the regions in Figure 2i.

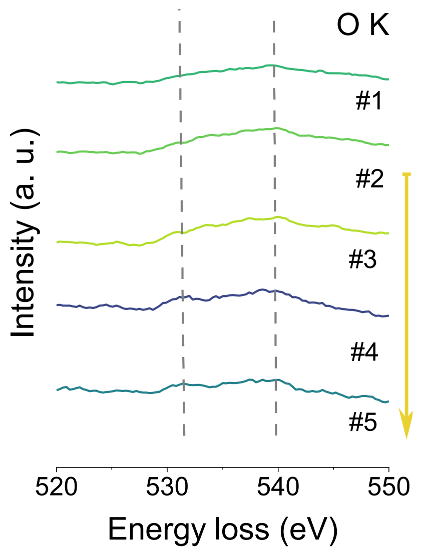

Figure S7. EELS spectra of O k-edge for different positions on L-CuNi in Figure 2i.

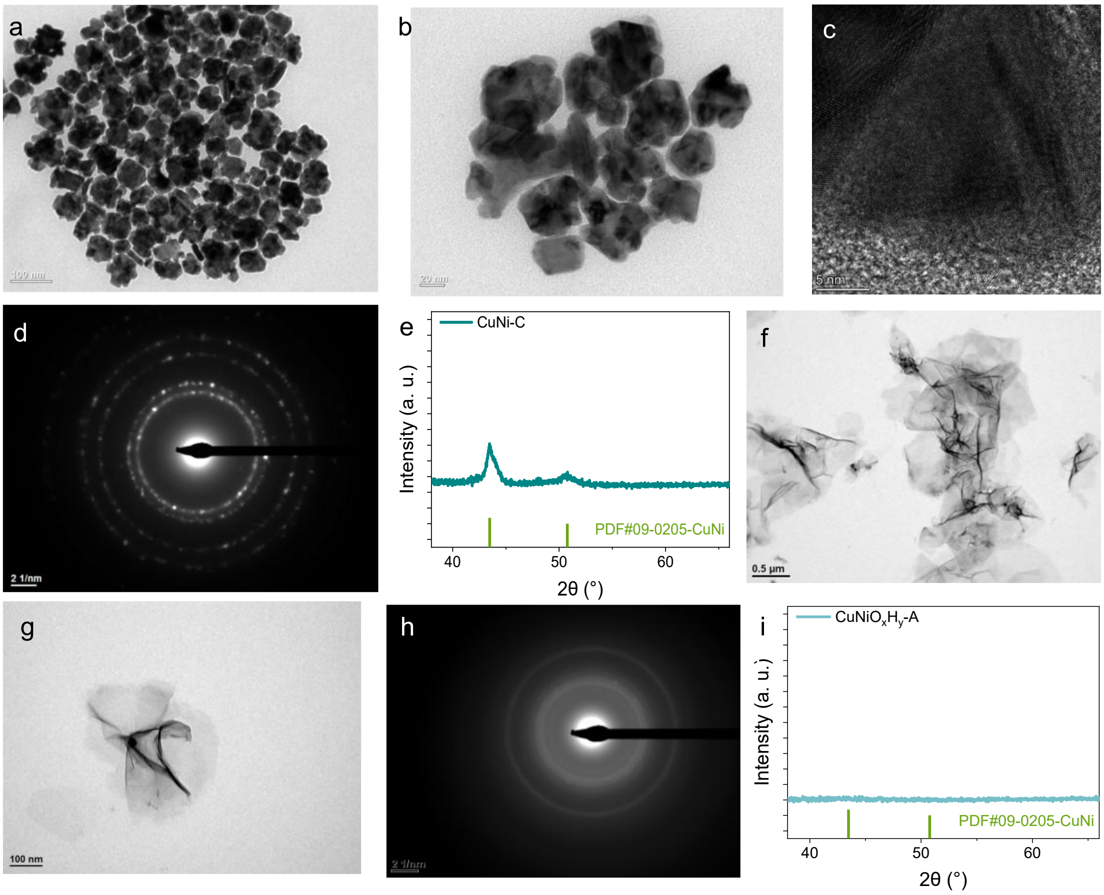

Figure S8. Structural characterization of CuNi-C and CuNiO$_x$H$_y$-A. (a,b) TEM images, (c) HR-TEM (d) FFT pattern and (e) XRD pattern of CuNi-C. (f-g) TEM images, (h) FFT pattern and (i) XRD pattern of CuNiO$_x$H$_y$-A.

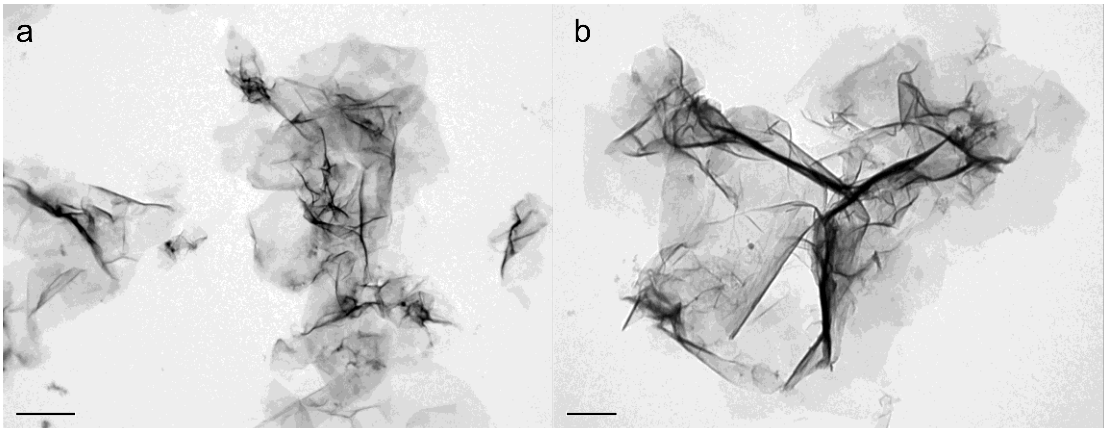

Figure S9. Structural characterization of $CuNiO_xH_y$-A. (a-d) TEM images of $CuNiO_xH_y$-A.

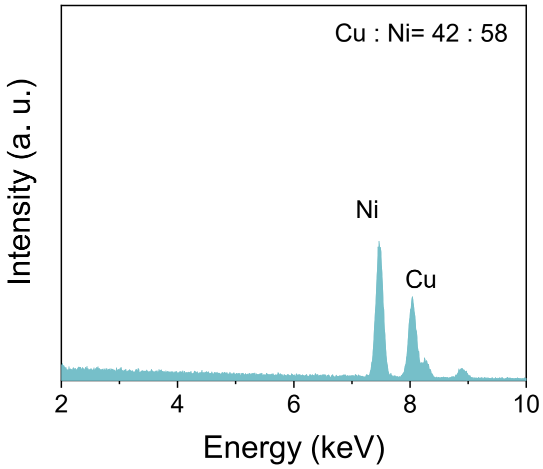

Figure S10. Composition characterization of CuNi-C. Typical EDS spectra of CuNi-C.

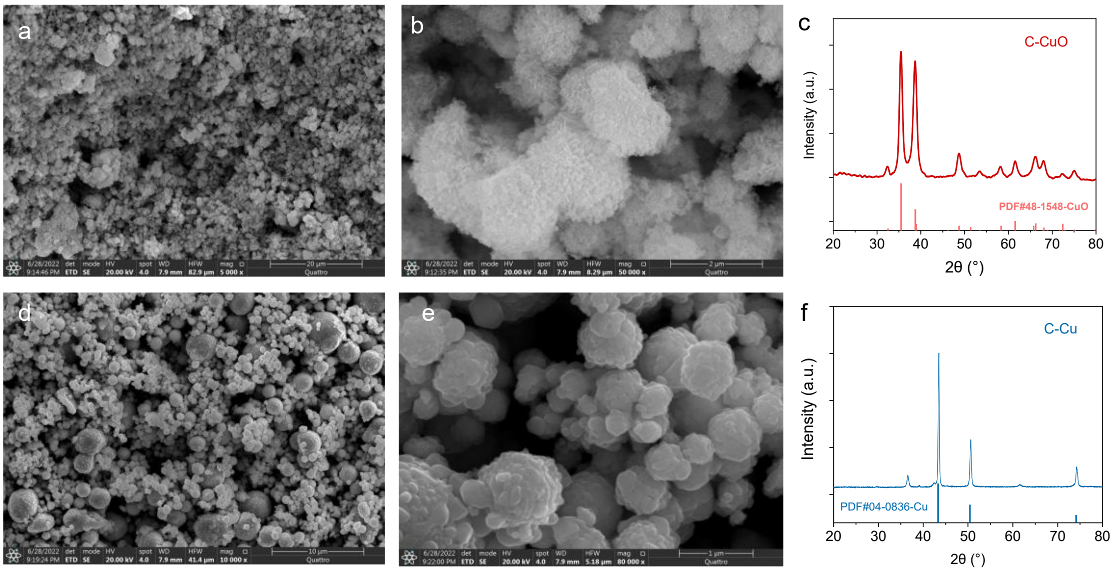

Figure S11. Structural characterization of C-CuO and C-Cu. (a, b) SEM images and (c) XRD pattern of C-CuO. (d, e) SEM images and (f) XRD pattern of C-Cu.

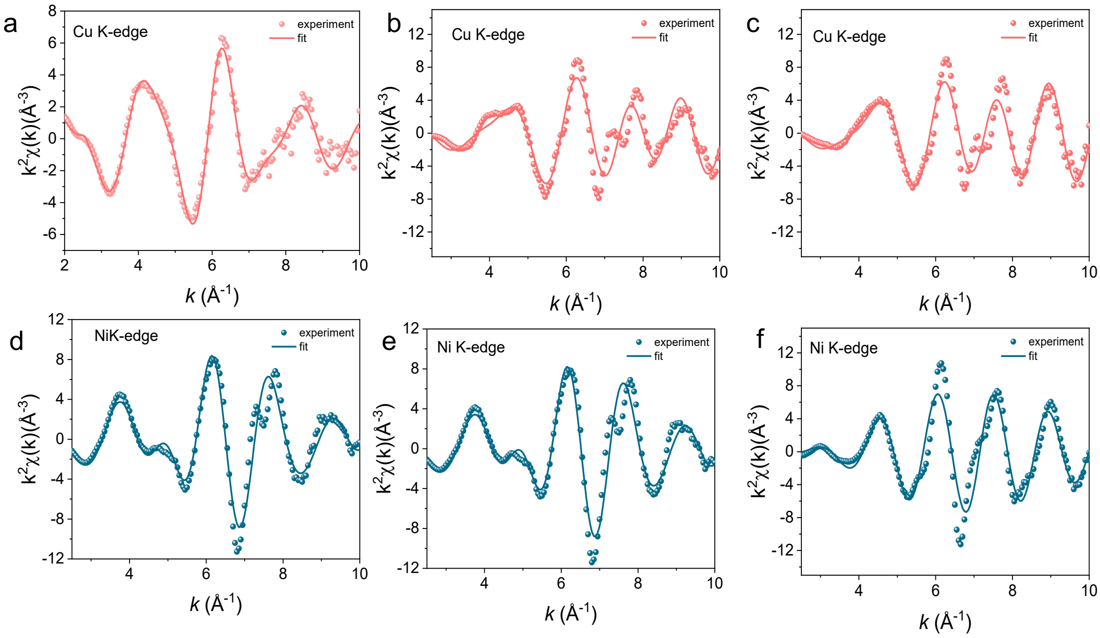

Figure S12. Fitting results of Cu K-edge and Ni K-edge EXAFS spectra of different catalysts. Fourier transform of Cu K-edge EXAFS fitting results of (a) L-CuNi, (b) CuNiO-A and (c) CuNi-C, respectively. Fourier transform of Ni K-edge EXAFS fitting results of (d) L-CuNi, (e) $CuNiO_xH_y$-A and (f) CuNi-C, respectively.

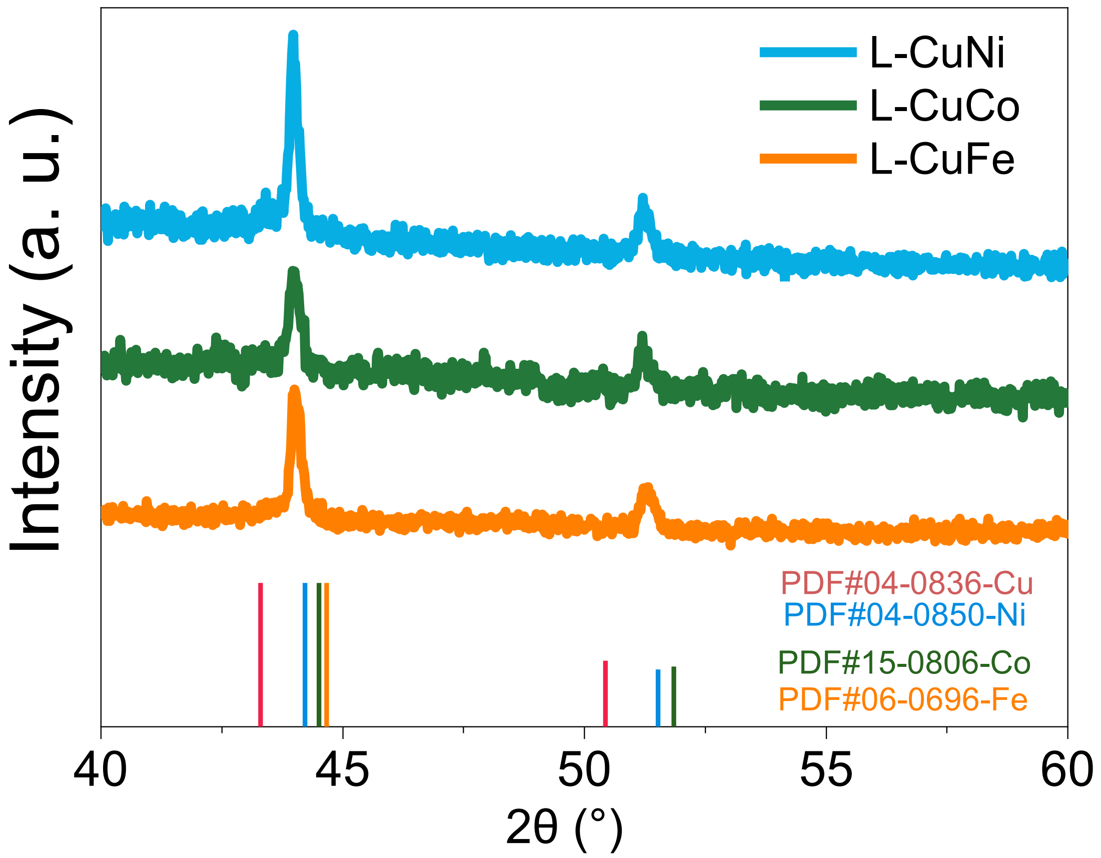

Figure S13. XRD pattern of L-CuNi, L-CuCo and L-CuFe. The XRD patterns show that the diffraction peak positions of the L-CuNi, L-CuCo, and L-CuFe samples all lie between those of the standard PDF cards for the corresponding elemental metals (Cu, Ni, Co, Fe), indicating the successful formation of Cu-based alloys. No apparent elemental metal phases are observed in the samples, and the primary crystalline structures of the three samples are highly similar.

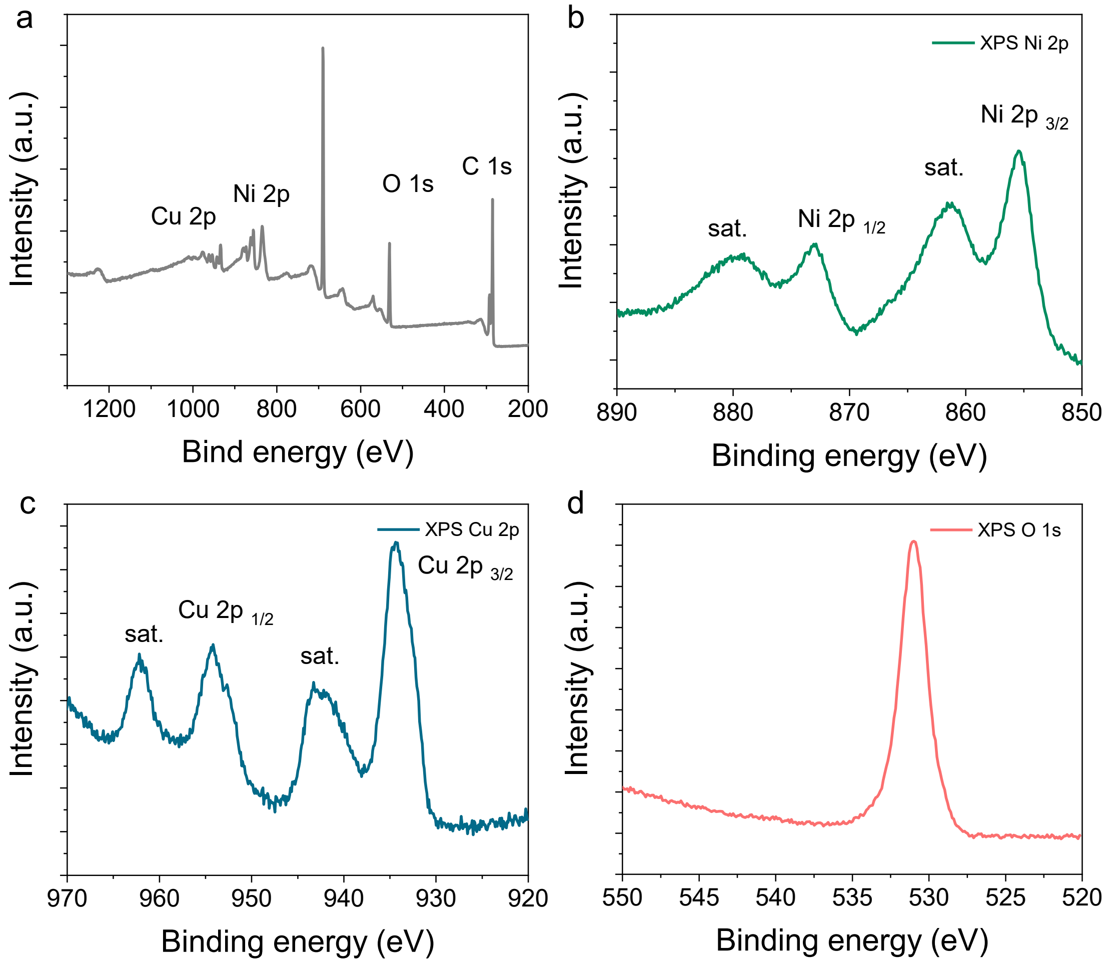

Figure S14. Structural characterization of L-CuNi. (a) Full spectrum (b) Ni 2p XPS spectra, (c) Cu 2p XPS spectra and (d) O 1s XPS spectra of L-CuNi.

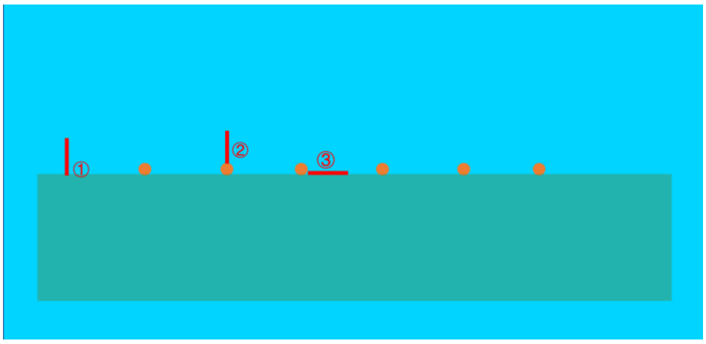

Figure S15. COMSOL simulation. Model design on different surface. (1) surface of $CuNiO_xH_y$-A (2) surface of CuNi-C (3) surface of L-CuNi. The electron density difference induced by the amorphous/crystalline two-phase interface leads to the formation of a built-in electric field near the spherical sites of the film. The electron density difference is represented by the different conductivity coefficients of the amorphous and crystalline phases.

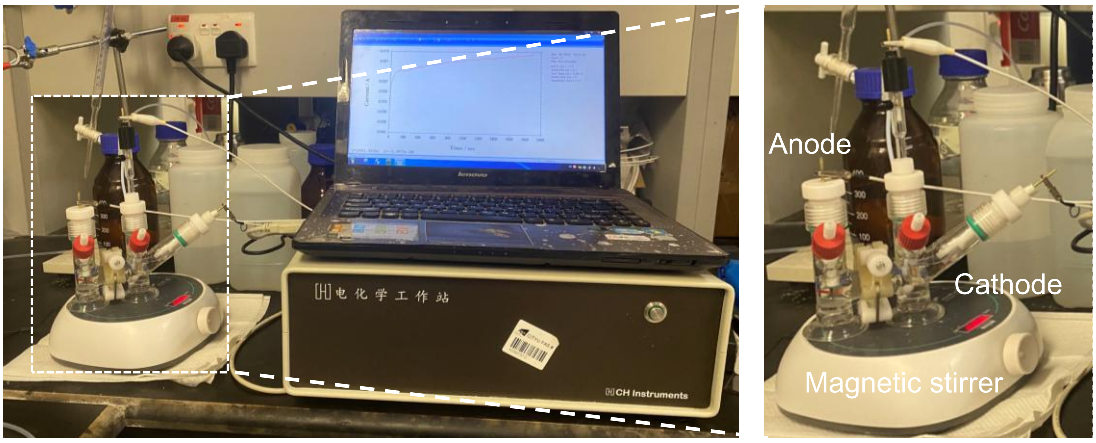

Figure S16. Digital image of NITRR device and corresponding partially enlarged image.

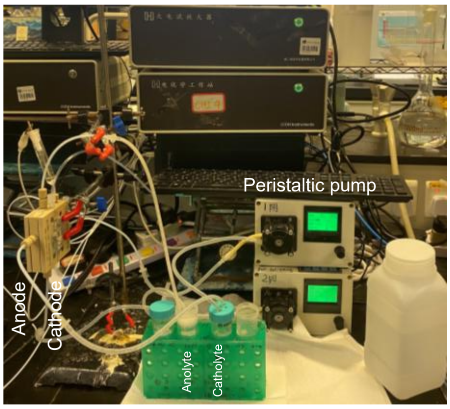

Figure S17. Digital image of Flow-Cell device for nitrate reduction.

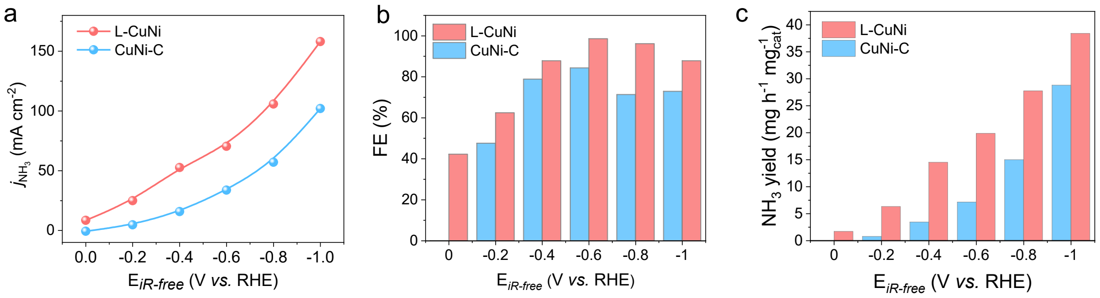

Figure S18. The comparation of partial current density, FE and NH_3 yield on L-CuNi and CuNi-C in 1 M KOH and 0.1 M KNO_3.

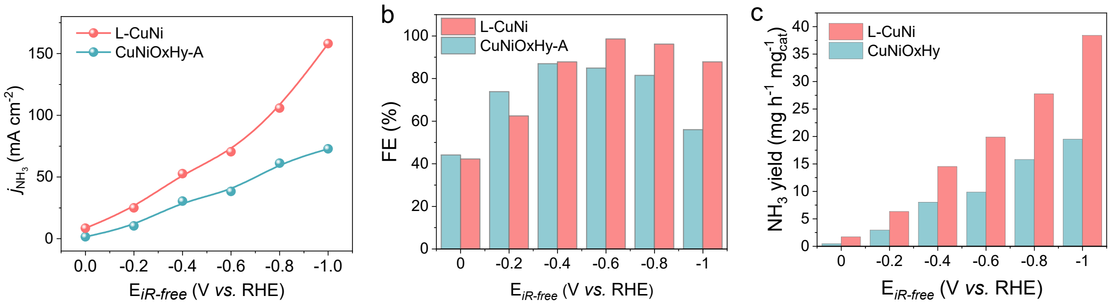

Figure S19. The comparation of partial current density, FE and NH_3 yield on L-CuNi and $CuNiO_xH_y$-A in 1 M KOH and 0.1 M KNO_3.

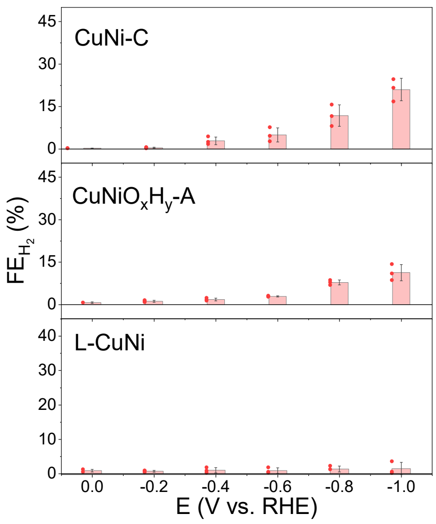

Figure S20. Faradaic efficiency of hydrogen in L-CuNi, $CuNiO_xH_y$-A and CuNi-C on different potentials, respectively.

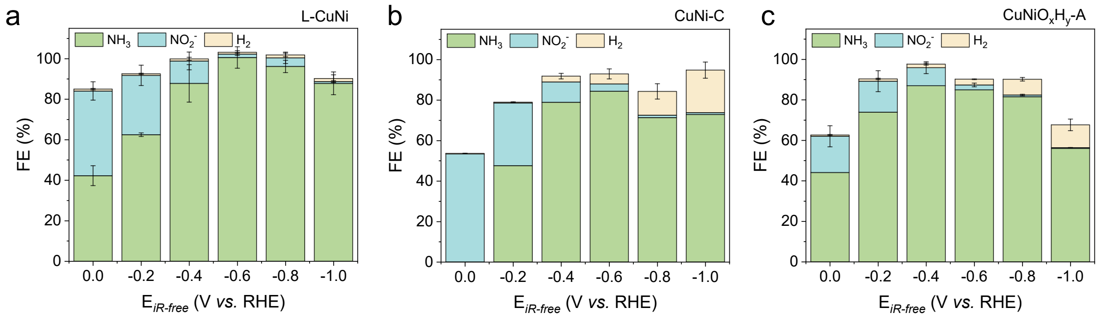

Figure S21. FE of observed products in L-CuNi (a), CuNi-C (b) and $CuNiO_xH_y$-A (c), respectively.

The result of L-CuNi shows a high NITRR performance in a wide range of overpotentials, especially in higher overpotential, compared with CuNi-C and $CuNiO_xH_y$-A. Although CuNi-C has excellent nitrate catalytic performance, at high potentials, due to the excessive hydrogen absorption capacity of nickel, HER gradually shows its advantages at high potentials. Moverover, the amorphous phases CuNiO-A's lower conductivity causes higher voltage drop at high current density, which hampers the NITRR as reflected by the lowest FE(NH_3) at high overpotential among all the controlled samples.

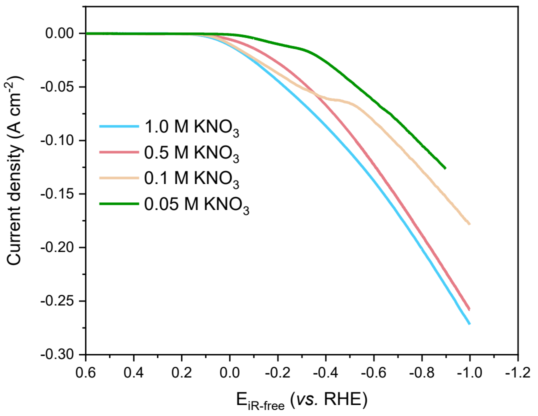

Figure S22. Linear scan voltammetry curves of L-CuNi in 1 M KOH + 0.05 M KNO_3, 1 M KOH + 0.1 M KNO_3, 1 M KOH + 0.5 M KNO_3 and 1 M KOH + 1 M KNO_3.

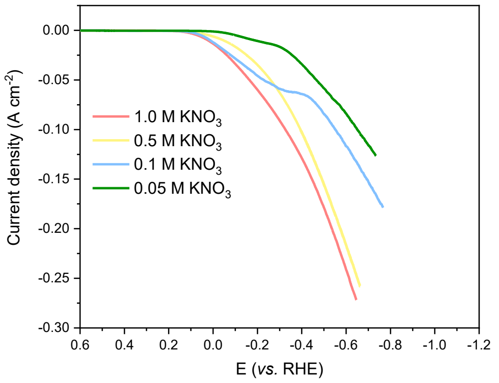

Figure S23. Linear scan voltammetry curves of L-CuNi in 1 M KOH + 0.05 M KNO_3, 1 M KOH + 0.1 M KNO_3, 1 M KOH + 0.5 M KNO_3 and 1 M KOH + 1 M KNO_3 with iR-80% compensation.

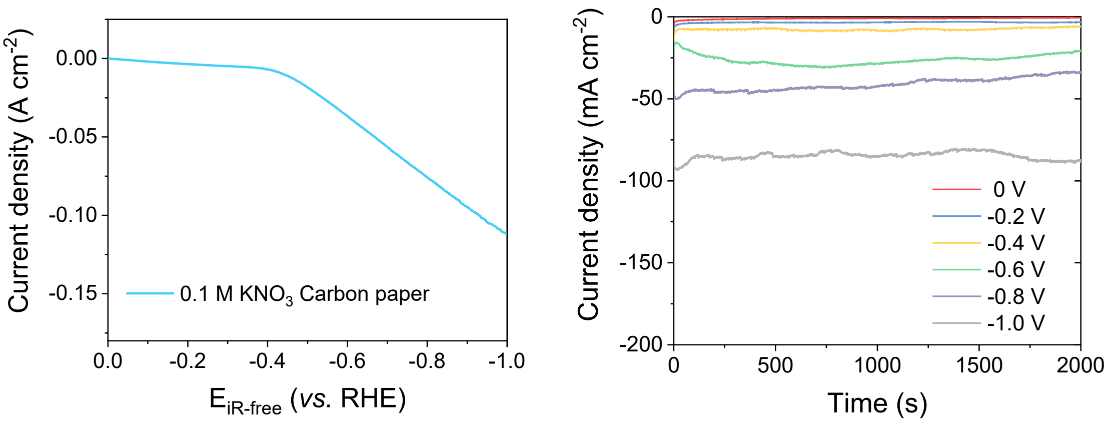

Figure S24. Electrochemical performances. (a) Chronoamperometry and (b) chronoamperometry on carbon paper at different potential in 1 M KOH + 0.1 M KNO_3.

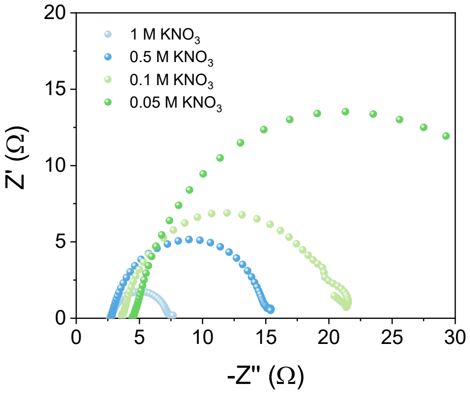

Figure S25. Nyquist plots in different electrolytes. L-CuNi in different electrolytes at 0 V vs. RHE.

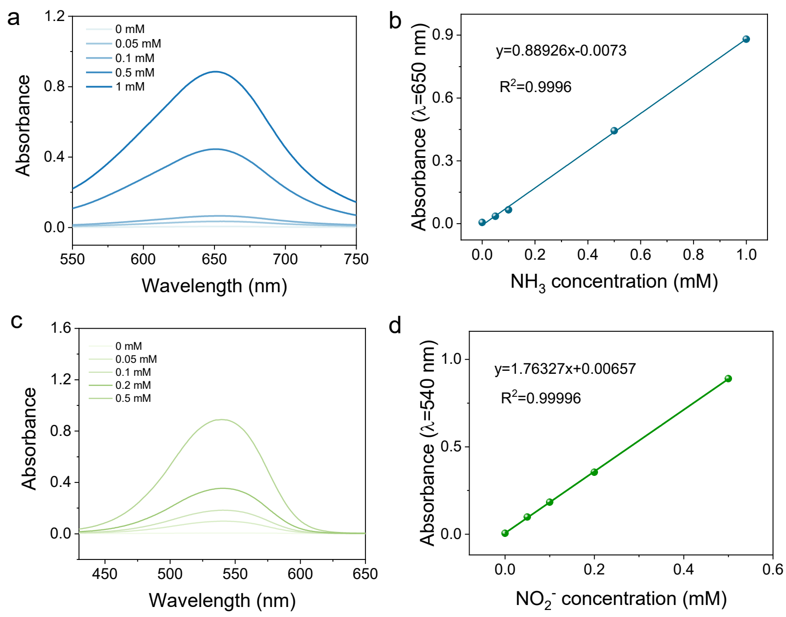

Figure S26. Calibration curves. UV-vis calibration curves of (a, b) ammonia (NH_3) and (c, d) nitrite (NO_2^-), respectively.

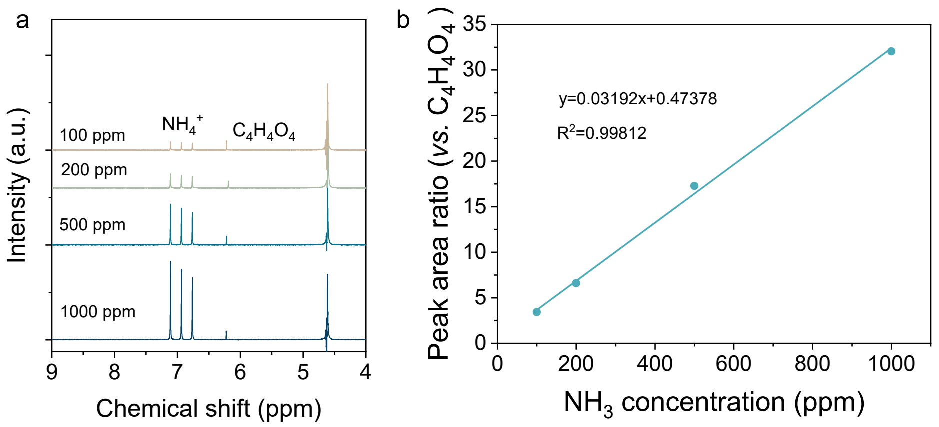

Figure S27. Calibration curves. (a) ^{1}H NMR calibration curve of NH_3 using different ammonium chloride concentration solutions of known concentration as standards. (b) ^{1}H NMR calibration curve of NH_3.

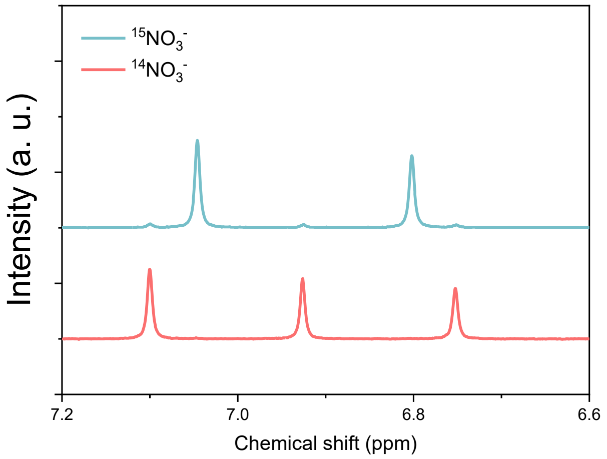

Figure S28. Representative 1H-NMR spectrum NMR spectra after NITRR using $^{15}NO_3^-$ and $^{14}NO_3^-$ electrolytes.

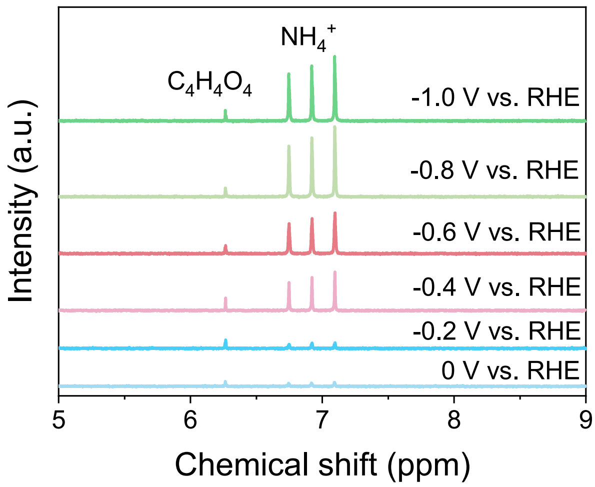

Figure S29. Product analysis. Representative 1H-NMR spectrum of liquid products collected from the cathode side.

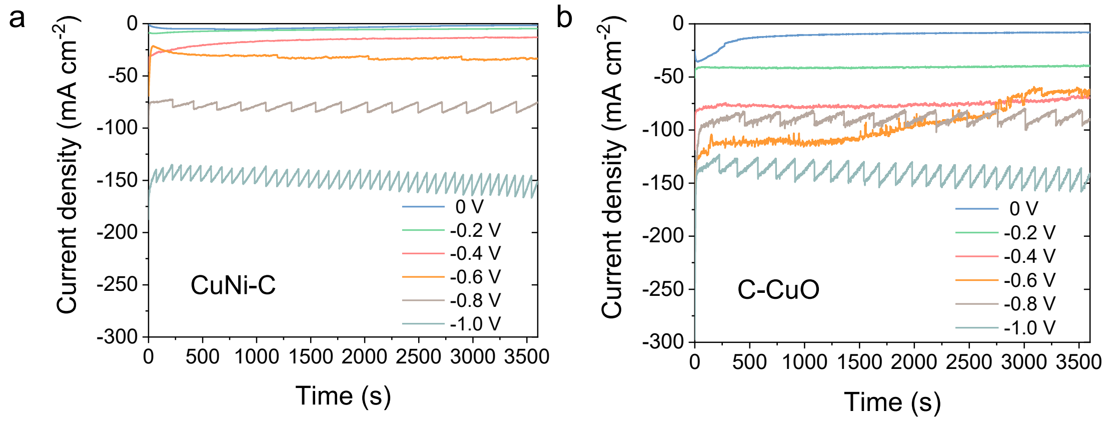

Figure S30. Chronoamperometry of (a) CuNi-C and (b) C-CuO in 1 M KOH + 0.1 M KNO_3.

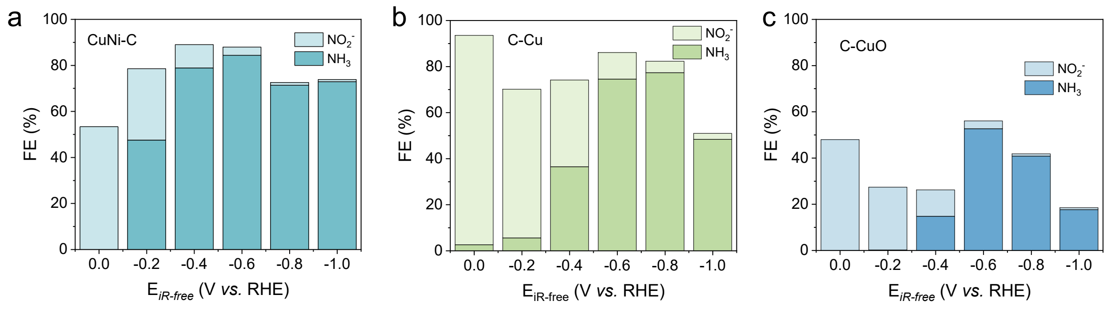

Figure S31. FE of NH_3 and NO_2^- of (a) CuNi-C, (b) C-Cu and (c) C-CuO in 1 M KOH + 0.1 M KNO_3.

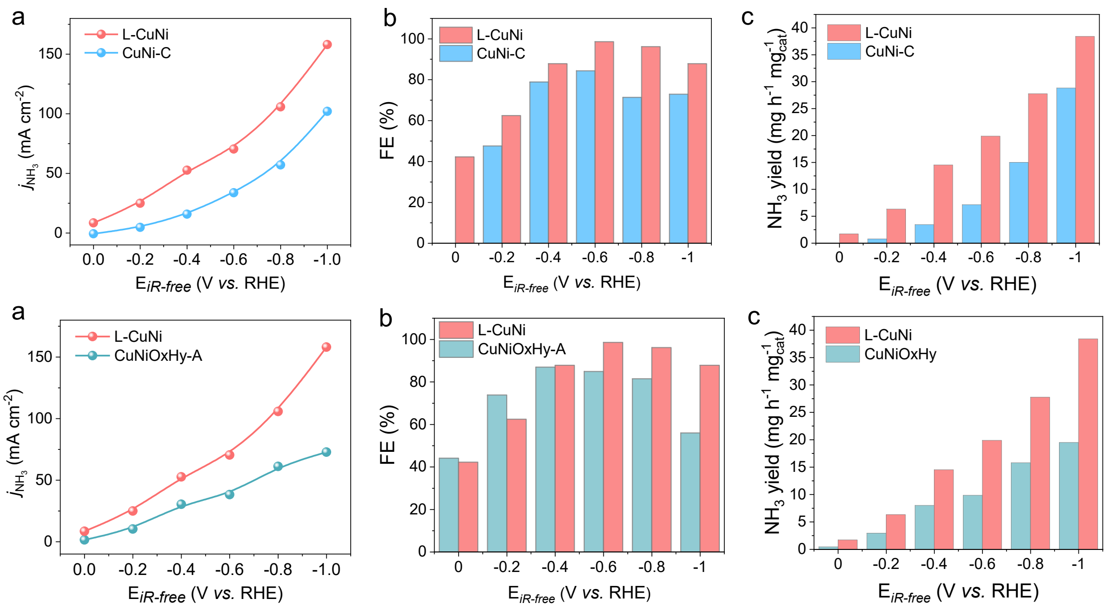

Figure S32. Combined NITRR performance. (a) NH_3 particle current density, (b) FE and (c) NH_3 yield of L-CuNi, CuNi-C and $CuNiO_xH_y$-A in 1M KOH +0.1 M KNO_3, respectively. (d-f) Chronoamperometry curves at different potentials of L-CuNi, $CuNiO_xH_y$-A and CuNi-C, respectively.

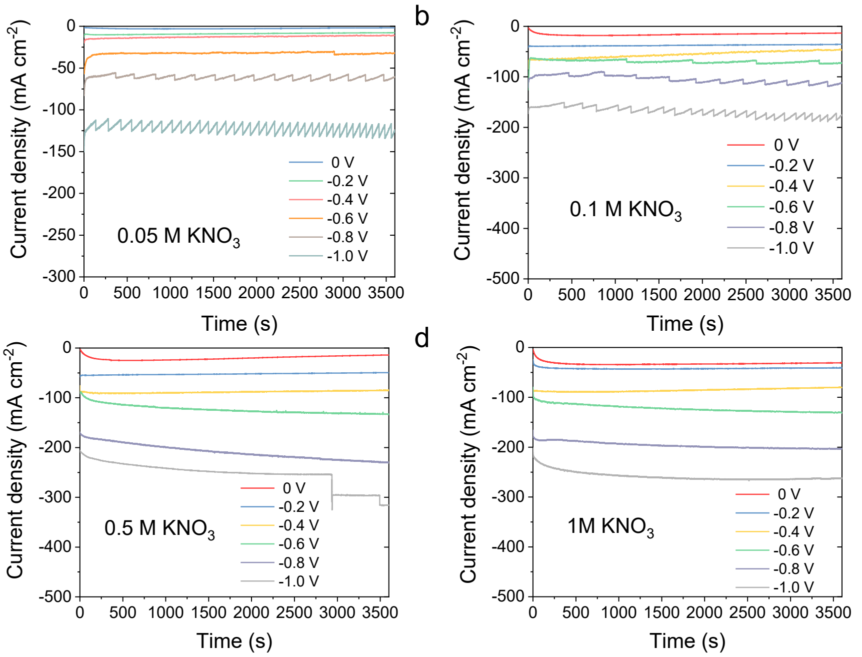

Figure S33. Electrochemical performances. Chronoamperometry of L-CuNi in (a) 1 M KOH + 0.05 M KNO_3, (b) 1 M KOH + 0.1 M KNO_3 and (c) 1 M KOH + 0.5 M KNO_3 and (d) 1 M KOH + 1 M KNO_3.

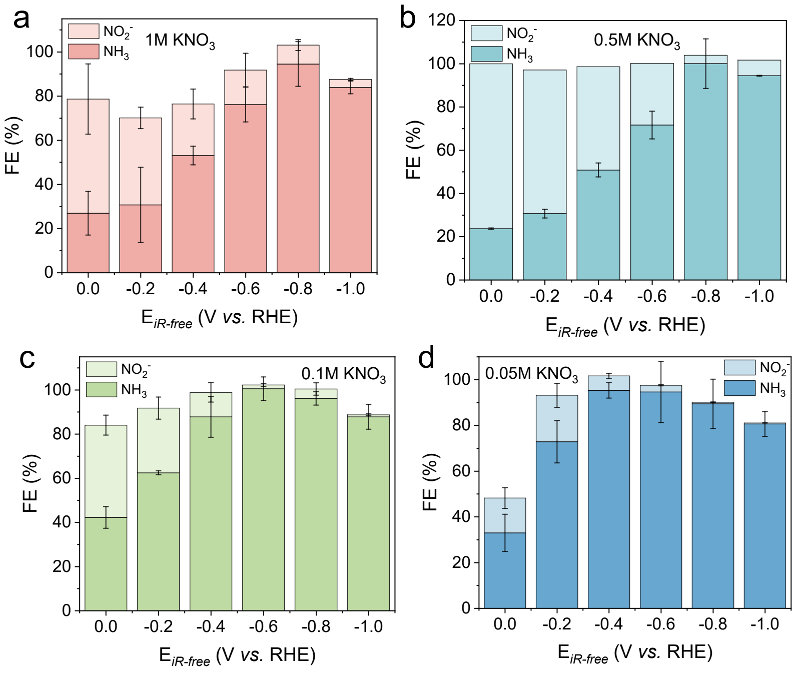

Figure S34. Electrochemical performances. FE of NH_3 and NO_2^- in (a) 1 M KOH + 1 M KNO_3 (b) 1 M KOH + 0.5 M KNO_3, (c) 1 M KOH + 0.1 M KNO_3 and (d) 1 M KOH + 0.05 M KNO_3.

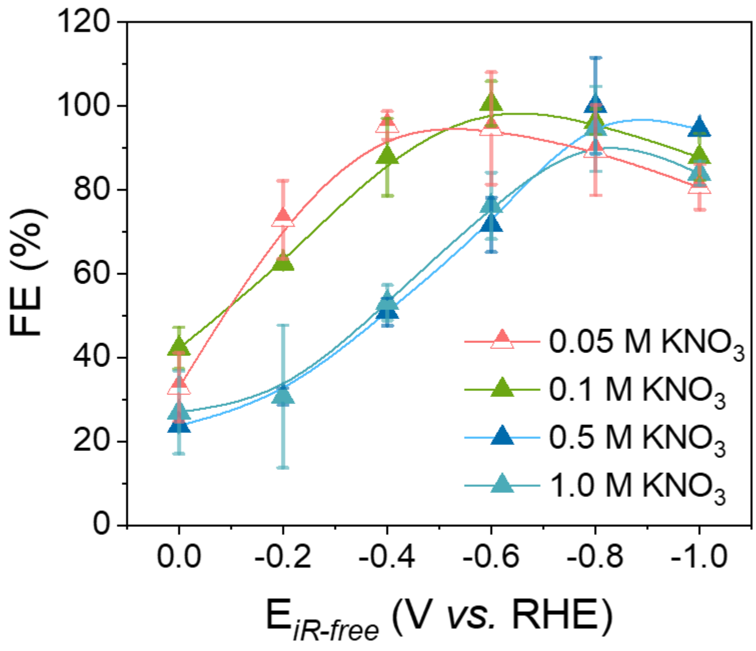

Figure S35. FE of NH_3 of L-CuNi in different concentrations of potassium nitrate.

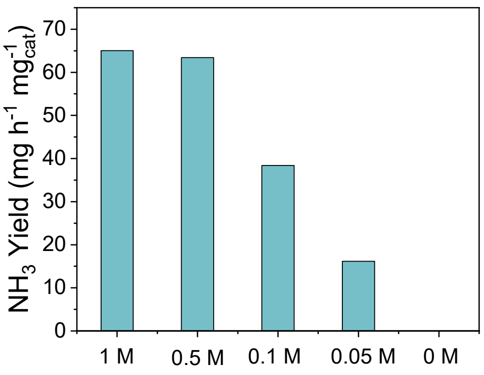

Figure S36. NH_3 yield of L-CuNi in 1 M KOH with 1 M KNO_3, 0.5 M KNO_3, 0.1 M KNO_3, 0.05 M KNO_3 and without KNO_3.

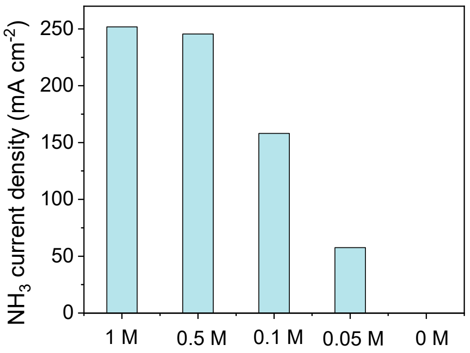

Figure S37. Electrochemical performances. NH_3 current density of L-CuNi in 1 M KOH with 1 M KNO_3, 0.5 M KNO_3, 0.1 M KNO_3, 0.05 M KNO_3 and without KNO_3.

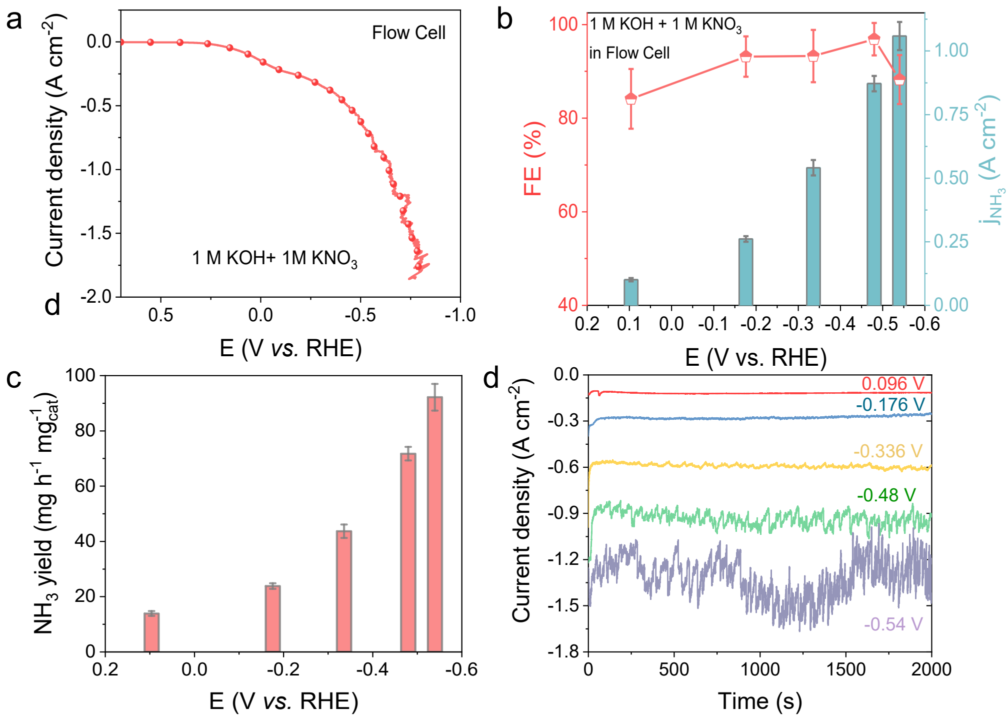

Figure S38. NITRR performance in flow cell. (a) Line scan voltage of L-CuNi during NITRR with 60% IR compensation. (b) FE (lift), j_{NH3} (right) (c) NH_3 yield and (d) Chronoamperometry curves at different potentials of L-CuNi in 1M KOH + 1M KNO_3 in flow cell during NITRR, respectively.

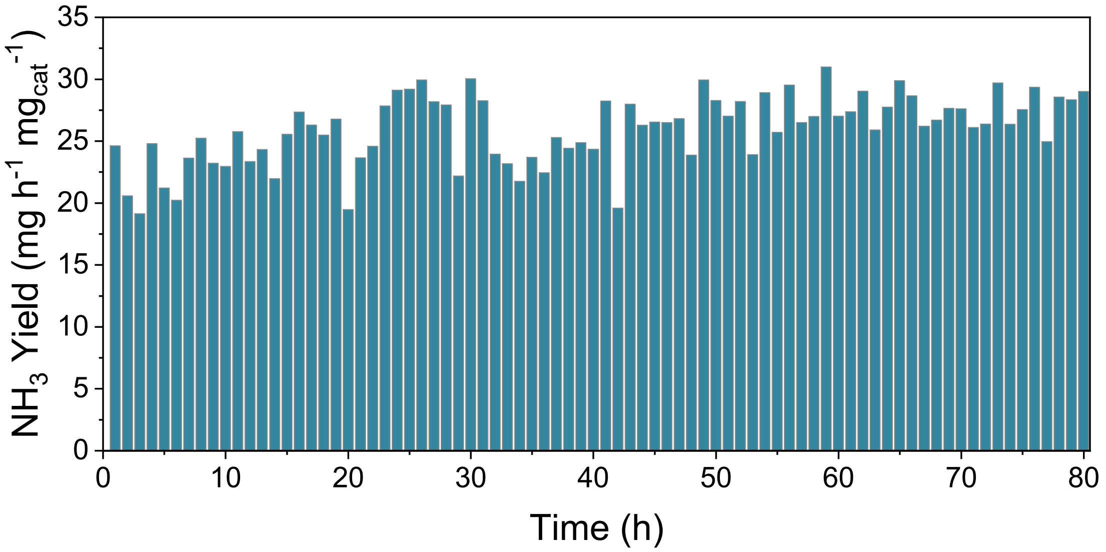

Figure S39. Electrochemical performances. NH_3 Yield of L-CuNi in 1 M KOH +0.1 M KNO_3 for 80h.

Up to 80 hours of stability of L-CuNi performance is mainly due to the unique structure of amorphous/crystal, which makes the catalyst structure flexible, allowing the materials to self-regulate and withstand structural disturbances during electrocatalysis,

improving their stability concerning long-term operations. For example, it was found that L-CuNi partially transforms from amorphous CuNiO to crystalline CuNi during the electrochemical process. This amorphous/crystalline catalyst generally has good stability test performance during the electrochemical test, demonstrated by many recent articles[1] Secondly, since we refresh the electrolyte every hour, the catalyst would experience oxidation and reduction repeatedly, accelerating the degradation of L-CuNi. Third, after an 80 h stability test, the Nafion binder in the catalyst will gradually relax, affecting the catalyst performance. This would induce the catalyst to dissolve partially and the current density to decrease. Compared with other literature, 80 hours of stability is relatively high, so we stopped the test.

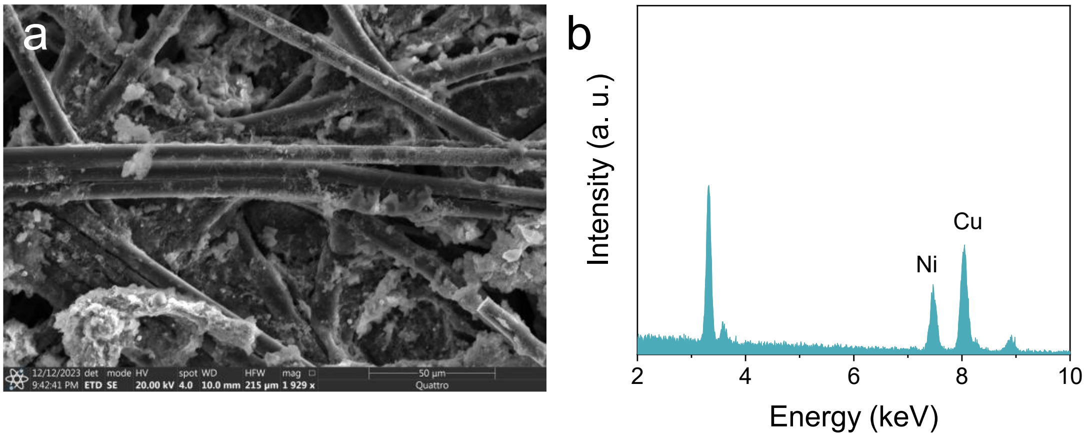

Figure S40. Composition characterization of L-CuNi. (a) SEM and (b) The EDS spectra of L-CuNi after 80 h stability.

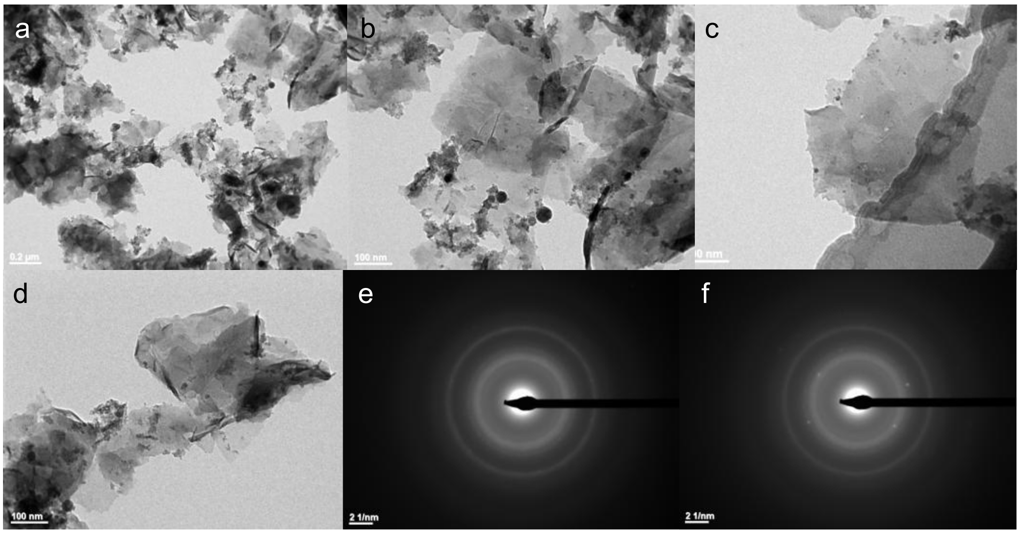

Figure S41. Composition characterization of L-CuNi. (a-d) TEM images and (e-f) The FFT pattern of L-CuNi after 80 h stability which indicates that the amorphous morphology is well maintained..

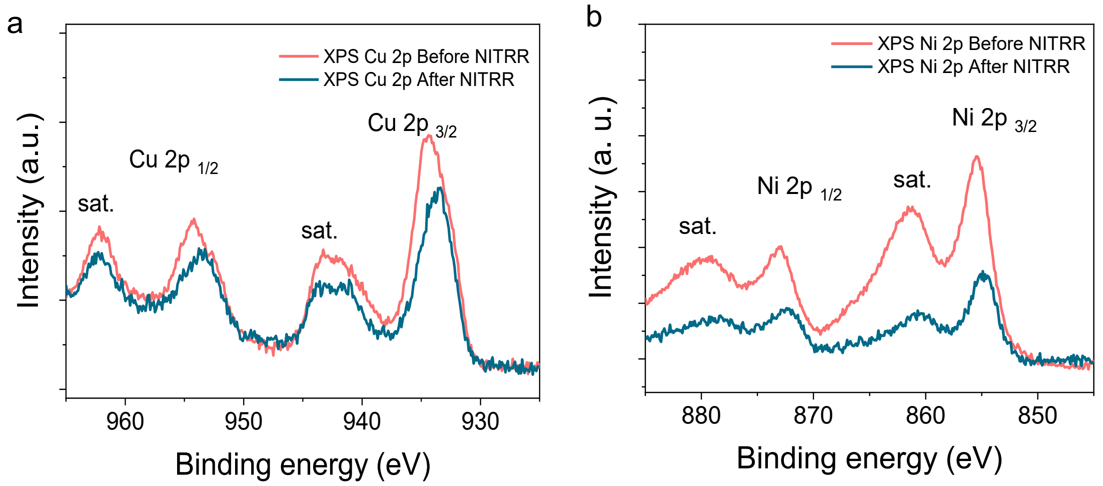

Figure S42. Characterizations of the tested L-CuNi. (a) Cu 2p XPS spectra and (b) Ni 2p XPS spectra of L-CuNi before and after stability test.

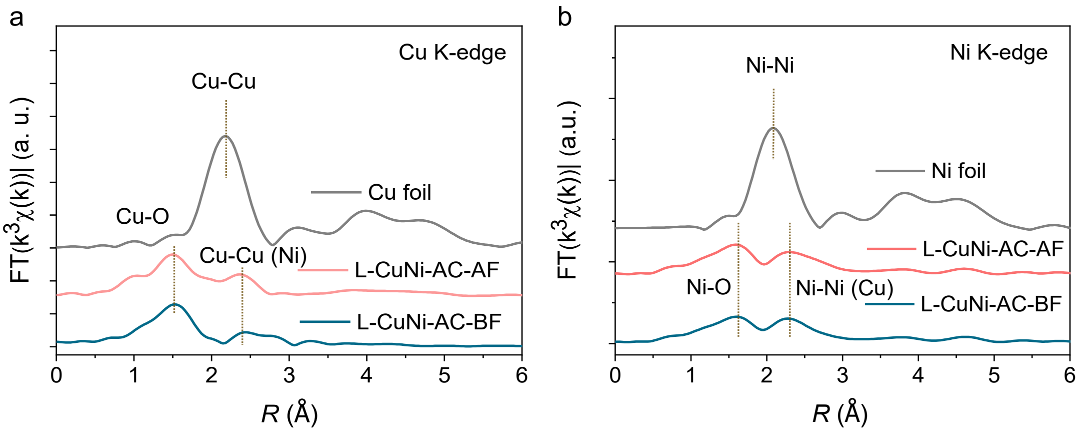

Figure S43. Characterizations of the tested L-CuNi. (a) Cu K-edge and (b) Ni K-edge of Fourier transformed EXAFS of L-CuNi before and after reaction.

We have characterized our post-reaction catalysts, including TEM, SEM, XPS, and the XANES test of post-catalyst. According to the above characterizations, in terms of morphology, the overall morphology of this catalyst is well preserved (SEM and TEM). Regarding the chemical state, the result of XPS spectra shows that the valence states of Cu and Ni are slightly reduced after the reaction. Still, both metals are predominantly in the oxidized state, as reflected by the satellite peaks. This result is consistent with the EXAFS data, showing similar coordination environments after the reaction.

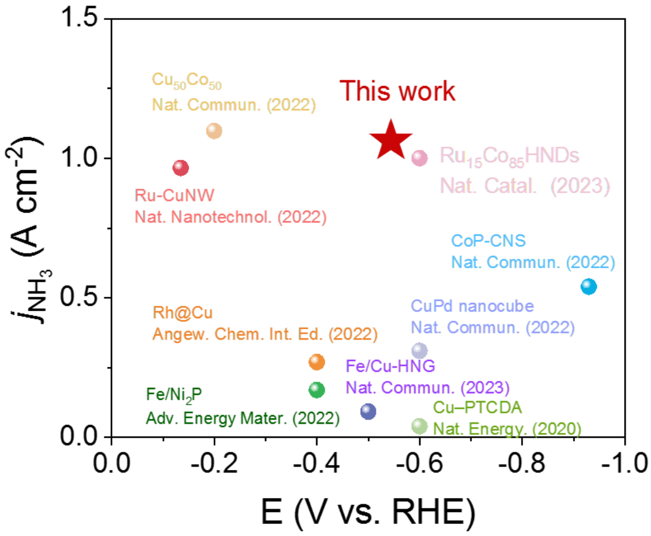

Figure S44. Comparation with that of state-of-the-art catalysts at a similar overpotential.

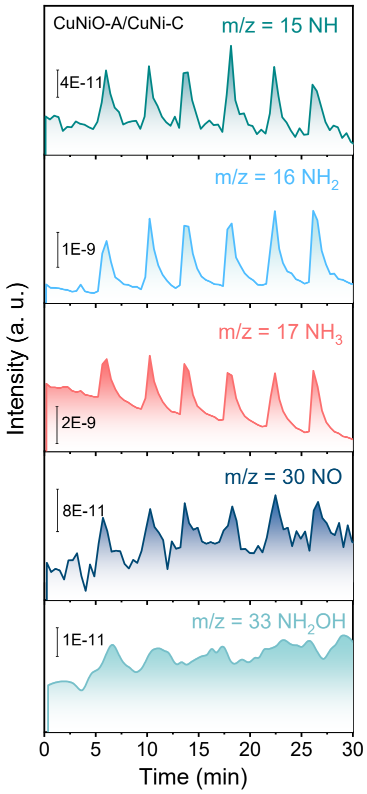

Figure S45. Online DEMS result. Online DEMS over L-CuNi.

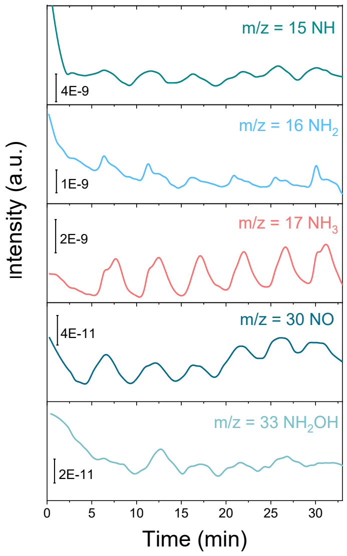

Figure S46. Online DEMS result. Online DEMS over C-CuO.

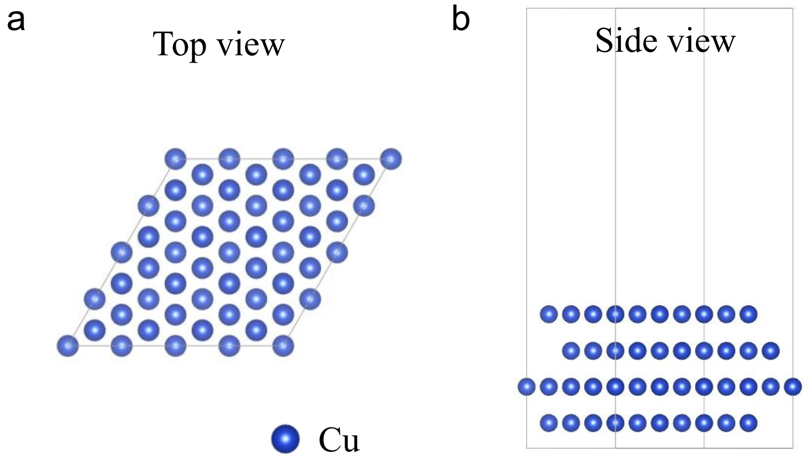

Figure S47. DFT simulation optimized geometry of Cu(111) slab at (a) top view and (b) side view.

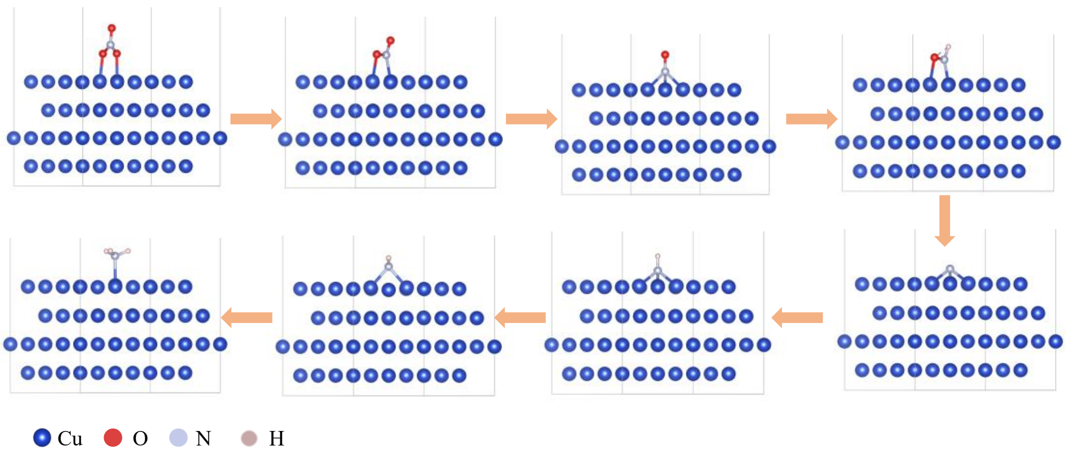

Figure S48. Optimized structures for the reaction intermediates of the NH_3 formation pathway on the Cu(111) slab.

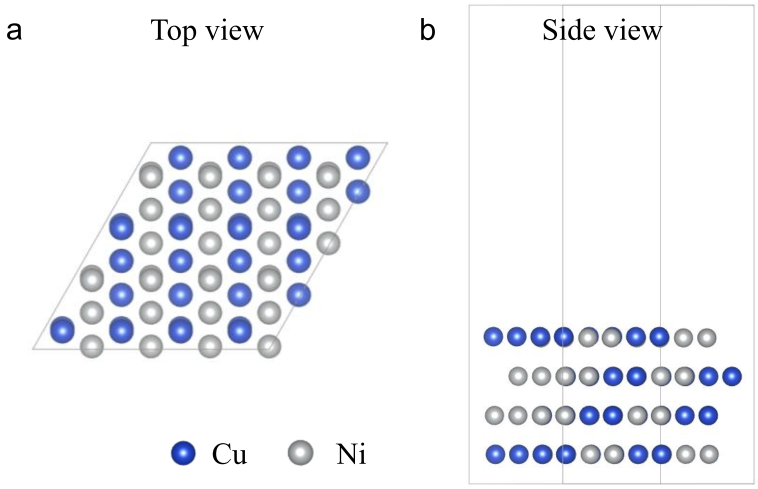

Figure S49. DFT simulation optimized geometry of CuNi(111) slab at (a) top view and (b) side view.

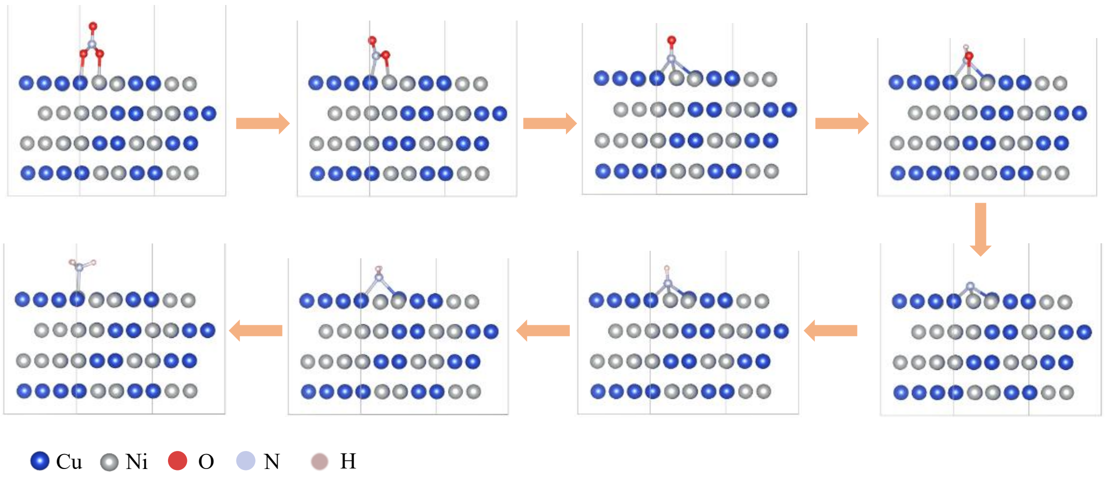

Figure S50. Optimized structures for the reaction intermediates of the NH_3 formation pathway on the CuNi(111) slab.

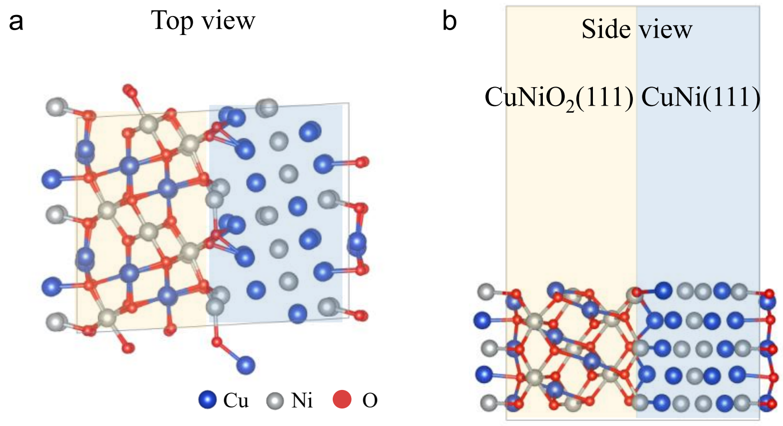

Figure S51. DFT simulation optimized geometry of L-CuNi(111) interface at (a) top view and (b) side view.

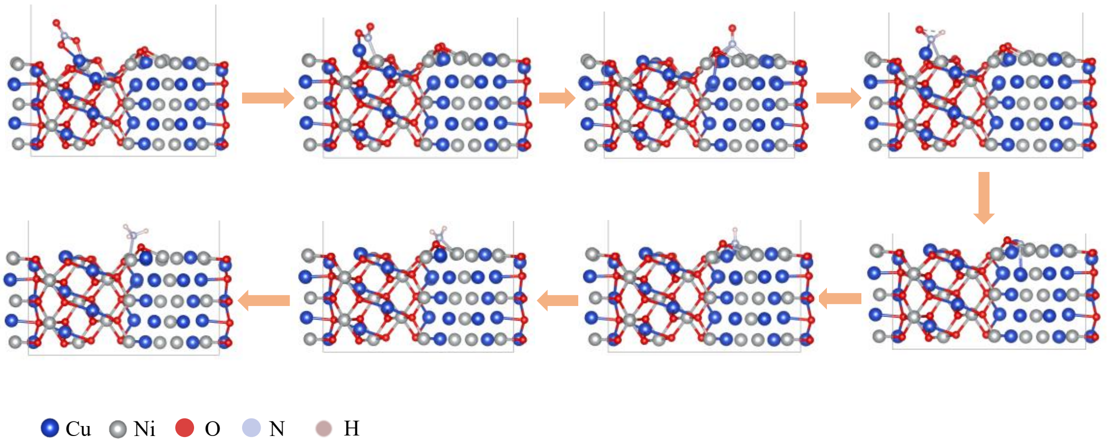

Figure S52. Optimized structures for the reaction intermediates of the NH_3 formation pathway on the $CuNiO_2$/CuNi(111) interface.

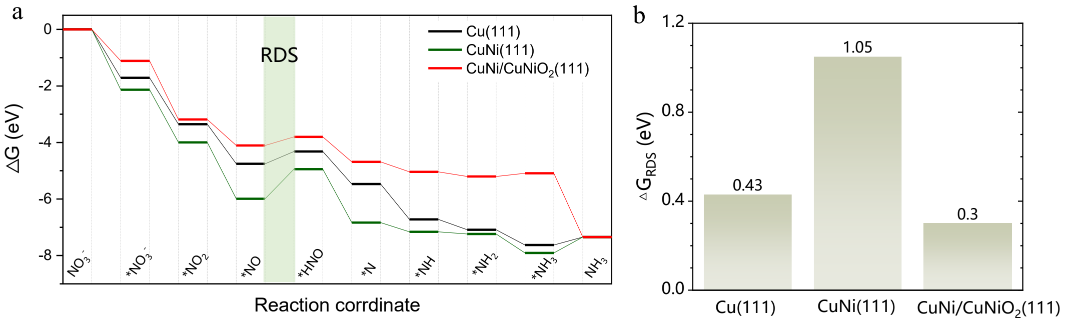

Figure S53. DFT simulation. (a) A reaction energy diagram for the NO_3^- to NH_3(g) on the Cu (111) slab, CuNi(111) slab, and $CuNiO_2$/CuNi(111) interface. (b) The free energy of the rate-determined step (RDS) for the HNO_3 to NH_3 pathway and the adsorption energy of the *HNO intermediate different slabs.

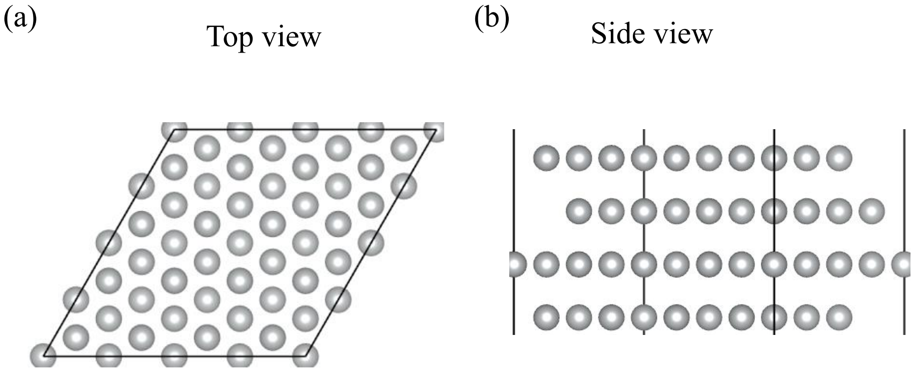

Figure S54. DFT simulation optimized geometry of Cu(111) slab at (a) top view and (b) side view.

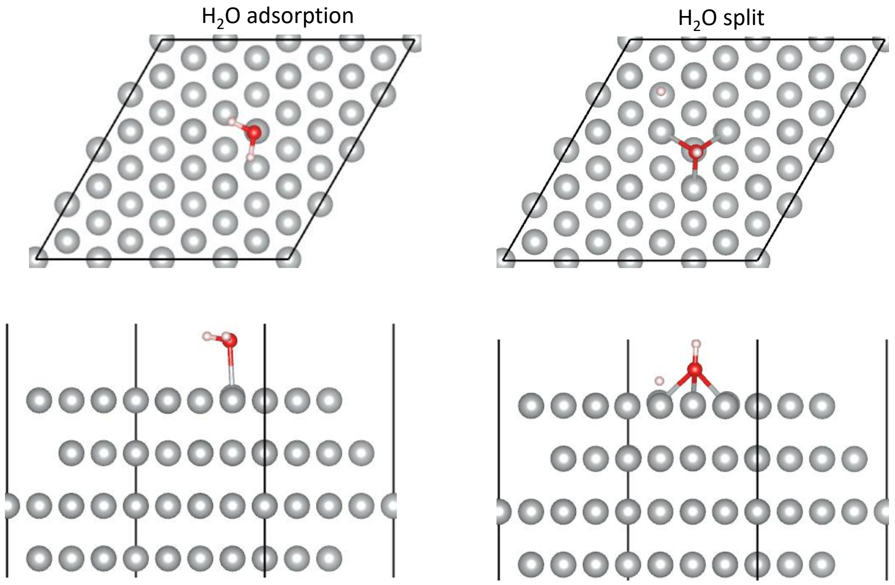

Figure S55. DFT simulation optimized geometry of H_2O absorption and split on Cu(111) slab.

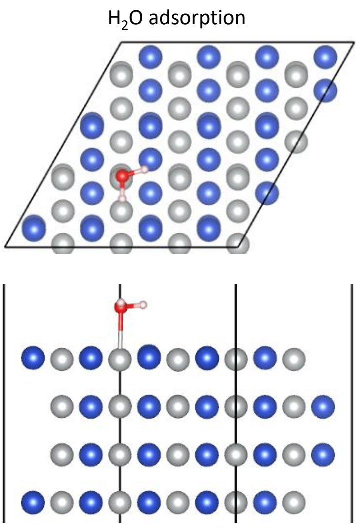

Figure S56. DFT simulation optimized geometry of H_2O absorption and split on CuNi(111) slab.

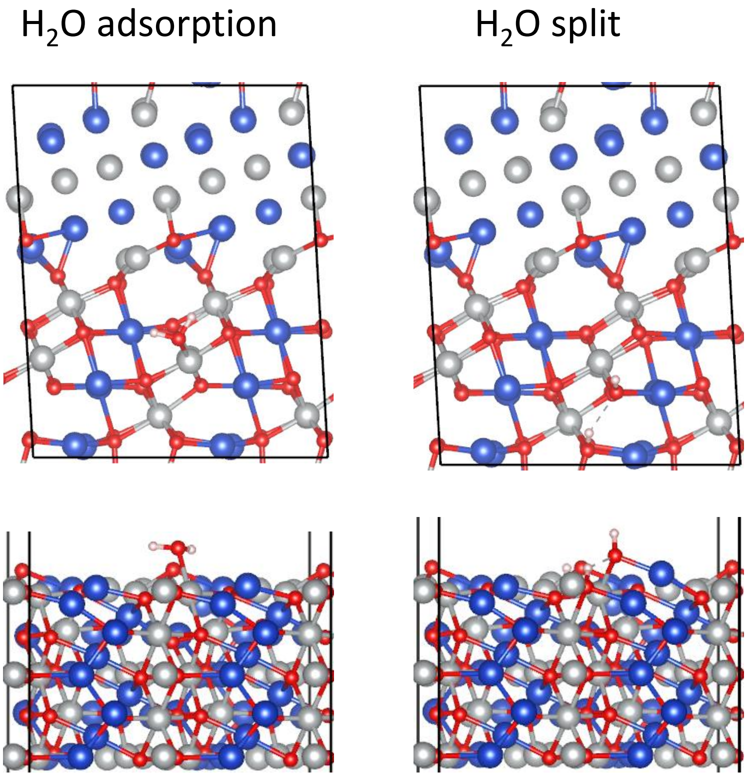

Figure S57. DFT simulation optimized geometry of H_2O absorption and split on $CuNiO_2$/CuNi(111) interface.

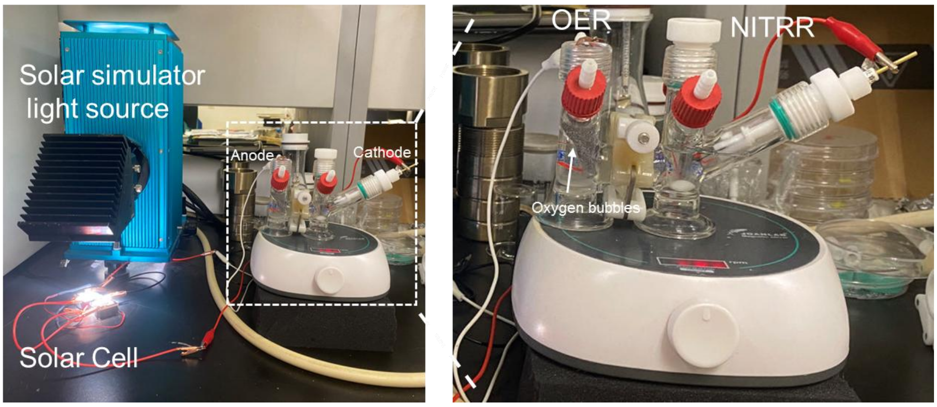

Figure S58. Digital image of solar-driven nitrate reduction device and corresponding partially enlarged image.

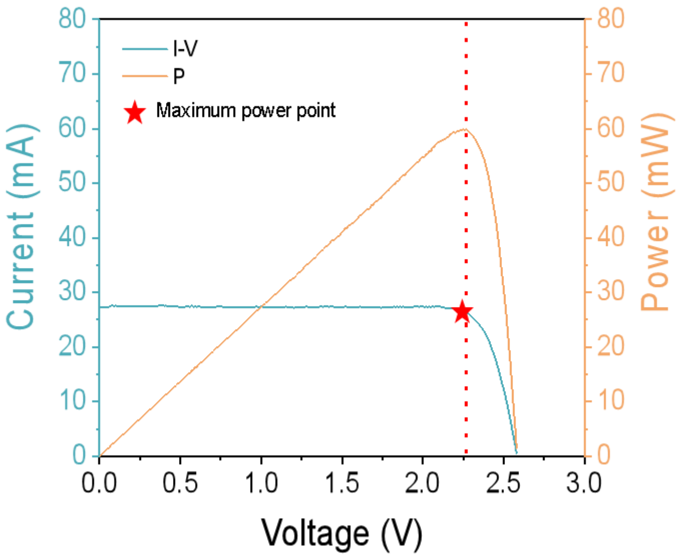

Figure S59. J-V characteristic of two GaInP/GaInAs/Ge triple junction cells connected in parallel.

The photovoltaic and electrochemical systems use solar cell to transform sunlight into electric power, which further drives electrochemical systems to produce ammonia. Hence, the solar cell's voltage and current need to match those in electrochemical systems for efficient energy utilization. The challenge comes from: (1) If the electrochemical system and solar system cannot match at the maximum power point (MPP) of the solar cell, it will result in a waste of solar energy. (2) In the whole-day operation, the sunlight intensity changes, resulting in fluctuation in solar-to-electricity energy efficiency. Thus, the catalysts should maintain a higher NITRR performance across a wide potential window.

Specifically, according to the J-V characteristic of two GaInP/GaInAs/Ge triple junction cells connected in parallel, the maximum solar-to-electric power conversion efficiency (PCE) of the photovoltaic is at 2.26 V-26.4 mA (PCE= 59.664 mW), as shown in Figure 60.

So, in order to get the maximum energy efficiency in solar-driven waste remediation, the electrochemical systems should be operated under the same voltage and current corresponding to MPP. Meanwhile, the FE of ammonia should be also high under this voltage and current.

In this situation, the solar-to-ammonia cell voltage value (V) can be divided into four parts:

$$V = E + \eta_{NITRR} + \eta_{OER} + iR$$

where E represents the standard thermodynamic cell potential for the conversion of nitrate and H_2O to ammonia and O_2, η_{NITRR} and η_{OER} represent the overpotentials for ammonia formation and oxygen evolution, and iR represents the ohmic loss between

two electrodes.

The η_{OER} generally starts after 1.23 V which has limited optimization space; therefore, it is necessary to obtain a catalyst with a wide window of high ammonia conversion to adjust η_{NITRR} to match the maximum power point (MPP) of the solar cell. The losses arising from a mismatch between the operating voltage and the voltage at the maximum power point (MPP) of the solar cell could be avoid.

However, the maximum power point (MPP) of photovoltaics reaches a high cell voltage of ~2.26 V, where most NITRR catalysts suffer from the competing side reactions in this high potential. The dominant hydrogen evolution at high overpotential further limits the working potential and decreases energy utilization efficiency.

Besides, since solar energy is easily affected by weather and time of the day, the solar to electric power conversion efficiency and the maximum power point of the solar cell will be affected. Thus, it is necessary to obtain a catalyst working efficiently across a wide potential window.

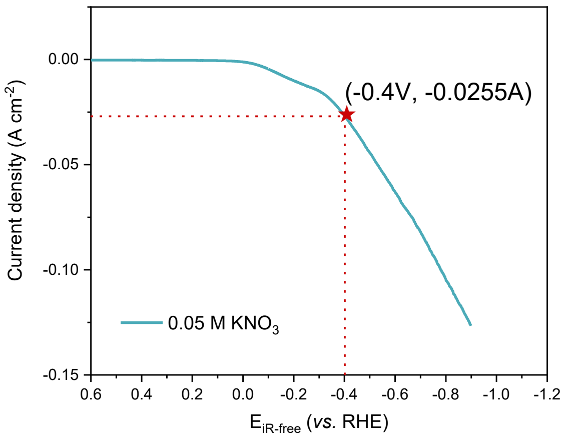

Figure S60. Solar to ammonia combined system. LSV of L-CuNi cathode in 1M KOH + 0.05 M KNO_3 and Ni foam anode.

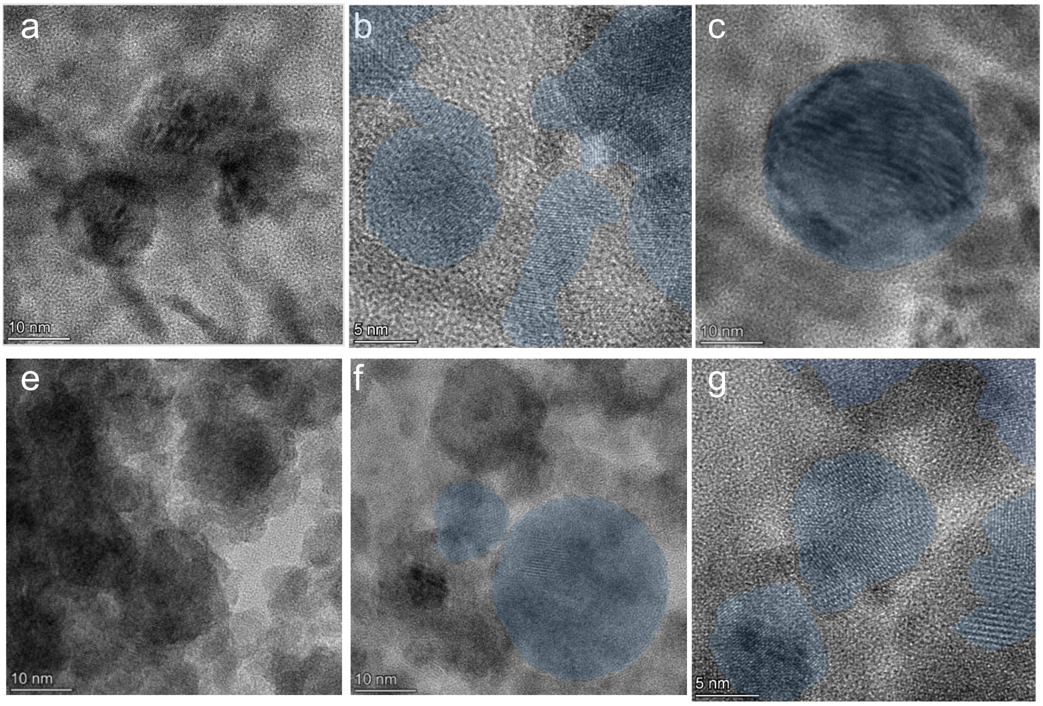

Figure S61. Structural characterization of L-CuCo and L-CuFe. (a-c) HR-TEM images of L-CuCo. (e-f) HR-TEM images of L-CuFe. Blue area: crystal particles.

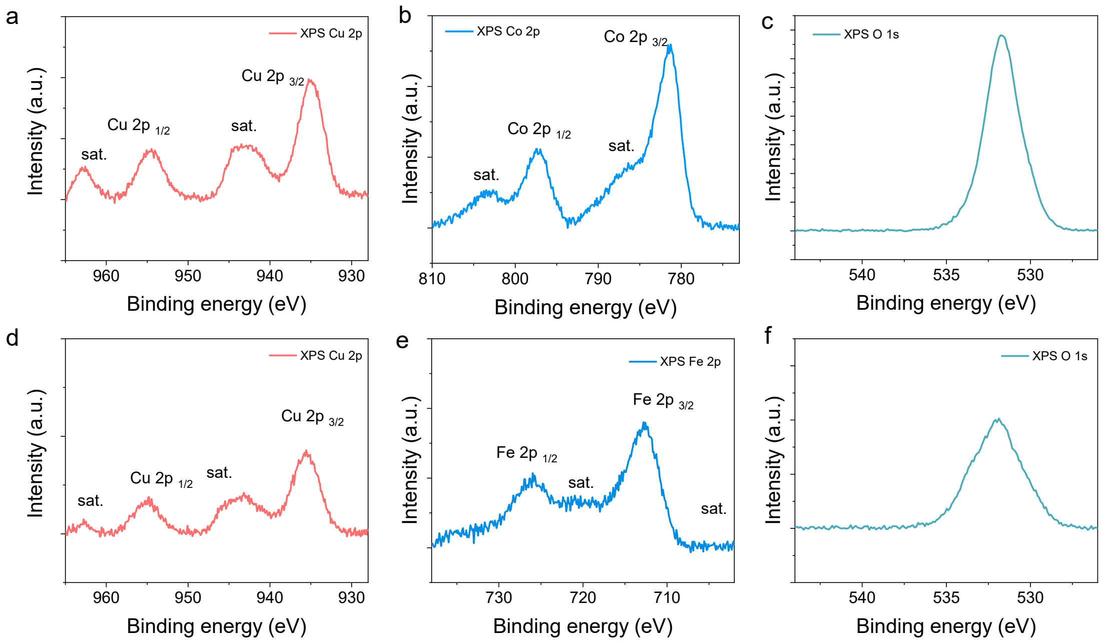

Figure S62. Structural characterization of L-CuCo and L-CuFe. (a-c) Cu 2p, Co 2p, and O 1s XPS spectra of L-CuCo. (e-f) Cu 2p, Fe 2p, and O 1s XPS spectra of L-CuFe.

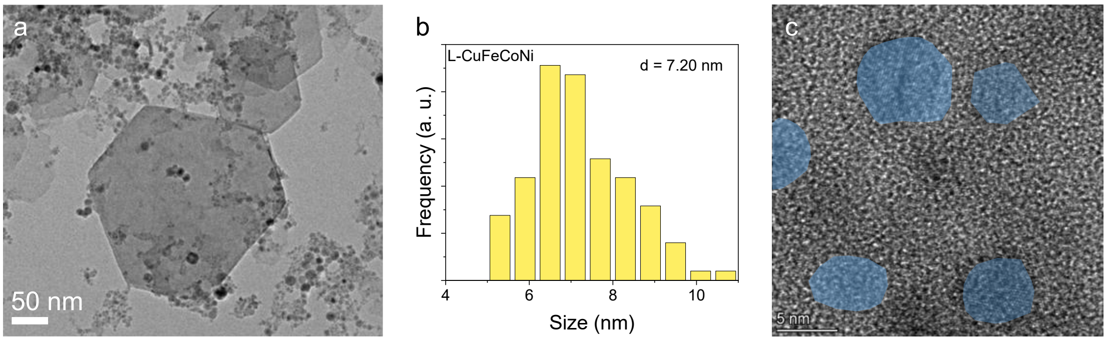

Figure S63. Structural characterization of L-CuFeCoNi. (a) TEM images. (b) Particle size statistics. (c) HRTEM image. Blue area: crystal particles.

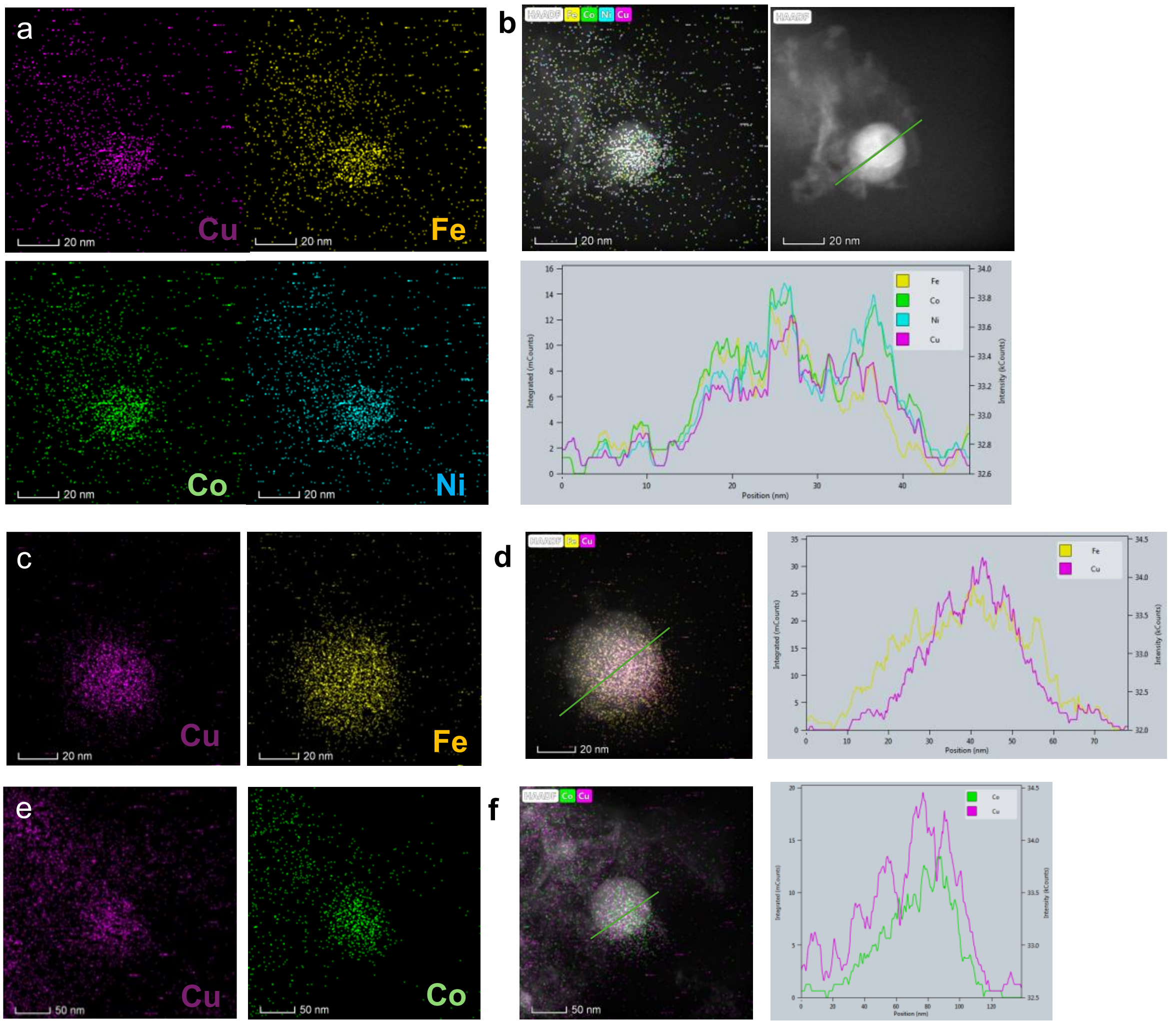

Figure S64. (a) EDX-mapping energy spectrum and (b) EDX line-scan of Cu, Fe, Co, Ni of L-CuFeCoNi. (c) EDX-mapping energy spectrum and (d) EDX line-scan of Cu, Fe of L-CuFe. (e) EDX-mapping energy spectrum and (f) EDX line-scan of Cu, Co of L-CuCo.

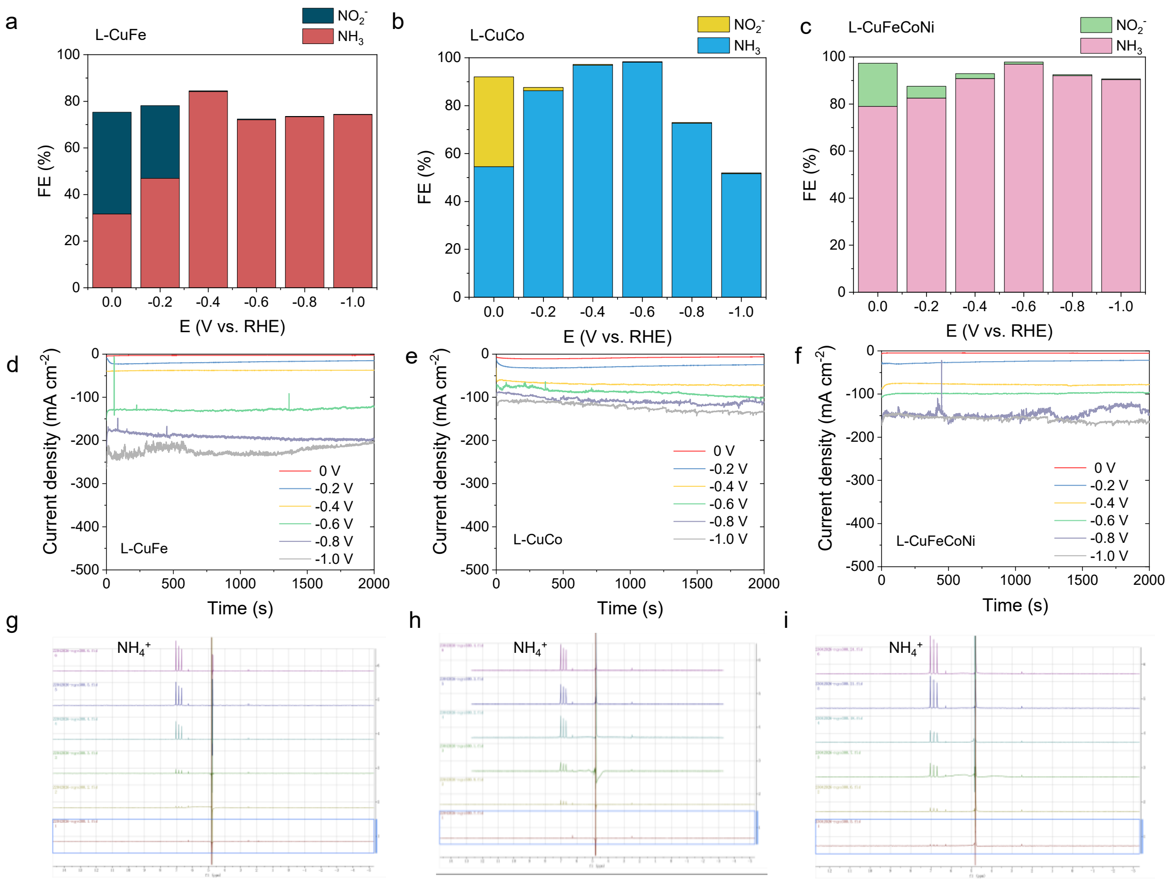

Figure S65. FE of NH_3 and NO_2^- of (a) L-CuFe, (b) L-CuCo and (c) L-CuFeCoNi, the corresponding chronoamperometry curve of (d) L-CuFe, (e)L-CuCo and (f) L-CuFeCoNi, the 1H-NMR spectrum of liquid products of (g) L-CuFe, (h)L-CuCo and (i) L-CuFeCoNi in 1 M KOH + 0.1 M KNO_3.

To validate the universality of the catalytic mechanism, we evaluated L-CuFe, L-CuCo, and L-CuFeCoNi for NITRR under the same conditions as L-CuNi. All three catalysts exhibited appreciable NH_3 yield rates and Faradaic efficiencies, with L-CuFeCoNi outperforming the binary systems. This confirms that the crystalline-amorphous heterostructure design is broadly applicable across multiple alloy/hydroxide combinations, and the catalytic trend is consistent with the electronic structure analysis.

Note S1

The techno-economic analysis was carried out using a modified model to calculate the total cost of production with units of US$ per ton of NH_3 via solar-to-NITRR, electricity-to-NITRR and NH_3 revenue price in 2023, respectively.

(1) For solar-to-ammonia: The total cost includes cost of solar panels, electrolyze cost, catalyst cost and product separation cost.

The price of electrolyzer is assumed to be 20000 \$ m^{-2}， our electrolyzer is 0.2*0.2 m^2, so costof electrolyzer is 20000*0.04=800 \$.[2]

The catalyst cost is assumed to be 10% of the electrolyzer cost[3]. The product separation cost is assumed to be 10% of the NH_3 revenue price in 2023.

The cost of solar panels is assumed to be 171 \$.

Thus, the total cost for solar-to-ammonia

= 800+80+123.7+171 = 1174.7 \$ t NH_3

(2) For electricity-to-ammonia: The total cost includes electricity cost, electrolyze cost, catalyst cost and product separation cost.

The price of electrolyzer , catalyst cost and the product separation cost are some with that in solar-to-ammonia systerm.

The electricity cost is assumed to the charge required to produce per ton NH_3.

The charge required to produce per ton NH_3:

$$Q = \frac{n(NH_3) \times N \times F}{FE(NH_3)} = \frac{m(NH_3) \times N \times F}{M(NH_3) \times FE(NH_3)} = \frac{1000000g \times 8 \times 96548}{17g\ mol^{-1} \times 90\%}$$
$$= 5.05 \times 10^{10} C$$

Where N is 8 electrons are required to convert one NO_3^- molecule to NH_3, $n(NH_3)$ is the total amount (in units of moles) of NH_3, F is the Faraday constant (F = 96485 C mol^{-1}), FE is the Faradaic efficiency of NO_3^- to NH_3.

The power required to sustain the process:

$$P = EI = \frac{EQ}{T} = \frac{2.2V \times 5.05 \times 10^{10}}{3600 \times 1000} = 3.08 \times 10^4\ kWh$$

Where E is the cell voltage.

The electricity cost = P × electricity price, when electricity price is reduced to 1 cents kWh^{-1}: Electricity cost = $3.08 \times 10^4 \times 0.01$= 308.00 \$ t_{NH3}^{-1}

Thus, the total cost for electricity-to-ammonia

= 800+80+123.7+308 = 1311.7 \$ t_{NH3}^{-1}

(3) The revenues can be calculated based on the market price of NH_3. The price of NH_3 is around 1237.00 \$ t^{-1}.[4]

The laser ablation in liquid (LAL) method employed in this study is currently a batch process with limited yield, which restricts its direct application for large-scale

production. Future work will focus on scalable strategies such as multi-beam parallel ablation or continuous-flow LAL systems to overcome this limitation.

Table S1. EXAFS fitting results of the Cu K-edge EXAFS for L-CuNi, CuNiO-A and CuNi-C, respectively.

Sample	path	CN	σ^2 ($Å^2$)	ΔE_0 (eV)	R (Å)	R-factor
L-CuNi	Cu-O	1.7	0.00816	0.008	1.97	0.014
	Cu-Cu	2.4	0.0046	0.0385	2.93	
	Cu-Cu	0.5	0.0037	0.0443	2.88	
CuNiO-A	Cu-O	2.9	0.0034	0.0024	1.95	0.008
	Cu-Cu	7.8	0.0089	-0.04212	2.93	
	Cu-Ni	1.8	0.0045	-1.76	2.56	
CuNi-C	Cu-Cu	3.9	0.00815	-1.2827	2.55	0.003

Table S2. EXAFS fitting results of the Ni K-edge EXAFS for L-CuNi, CuNiO-A and CuNi-C, respectively.

Sample	path	CN	σ^2 ($Å^2$)	ΔE_0 (eV)	R (Å)	R-factor
L-CuNi	Ni-O	3.3	0.0043	-0.054	2.05	0.008
	Ni-Ni	8.1	0.0086	0.047	3.02	
	Ni-Cu	1.5	0.0043	0.043	2.52	
CuNiO-A	Ni-O	3.1	0.0047	-0.057	2.05	0.007
	Ni-Ni	7.2	0.0176	0.037	3.01	
	Ni-Cu	1.4	0.0029	0.040	2.51	
CuNi-C	Ni-Cu	7.26	0.0118	0.0762	2.55	0.026

Table S3. Comparison of the NTIRR performance of the L-CuNi in this work with the previously reported Cu-based catalysts.

Catalysts	Electrolyte	Potential (V)	Ammonia Current density ($mA\ cm^{-2}$)	Ammonia Yield		Ref.
				$mg\ h^{-1}\ mg_{cat}^{-1}$	$mmol\ h^{-1}\ mg_{cat}^{-1}$	
**L-CuNi**	1 M NO_3^-<br>1 M KOH<br>(H-Cell)	-1.0 V<br>(*iR*-free)	251.8	65.01 $mg\ h^{-1}\ mg_{cat}^{-1}$	3.82 $mmol\ h^{-1}\ mg_{cat}^{-1}$	★**This work**
	1 M NO_3^-<br>1 M KOH<br>(Flow Cell)	-0.54 V	1058.9	92.18 $mg\ h^{-1}\ mg_{cat}^{-1}$	5.42 $mmol\ h^{-1}\ mg_{cat}^{-1}$	
**Fe/Cu-HNG**	1M KOH and 0.1M KNO_3	-0.5 V	92.51	N.A.	1.08 $mmol\ h^{-1}\ mg^{-1}$	5
**CuPd nanocube**	1 M NO_3^-<br>1 M KOH	-0.6 V	310	N.A.	6.25 $mmol\ h^{-1}\ mg^{-1}$	6
**Cu–PTCDA**	36 mM NO_3^-<br>0.1 mM PBS	-0.6 V	40	0.44 $mg\ h^{-1}\ cm^{-2}$	0.0256 $mmol\ h^{-1}\ cm^{-2}$	7
**$Cu_{50}Co_{50}$**	100 mM NO_3^-<br>1 M KOH	-0.2 V	1098	N.A.	4.58 $mmol\ h^{-1}\ cm^{-2}$	8
**Pd-Cl/Cu_2O**	1 M KNO_3 + 1 M KOH	-0.6 V	~2180	330 $mg\ h^{-1}\ cm^{-2}$	N.A.	9
**Rh@Cu**	100 mM NO_3^-<br>0.1 M Na_2SO_4	-0.4 V	270	N.A.	1.27 $mmol\ h^{-1}\ cm^{-2}$	10
**Ru-CuNW**	32 mM KNO_3 + 1 M KOH	–0.135 V	965	76.6 $mg\ h^{-1}\ cm^{-2}$	N.A.	11
**CoP-CNS**	1400 ppm NO_3^-<br>1 M KOH	-0.93 V	540	N.A.	3.093 $mmol\ h^{-1}\ cm^{-2}$	3
**Fe/Ni_2P**	500 mM NO_3^-<br>0.1 M K_2SO_4	-0.4 V	170	4.17 $mg\ h^{-1}\ cm^{-2}$	N.A.	12
**$Ru_{15}Co_{85}$HNDs**	1 M KNO_3 + 1 M KOH	-0.6 V	~1000	119 $mg\ h^{-1}\ cm^{-2}$	3.3 $mmol\ h^{-1}\ mg^{-1}$	13

Table S4. Summary of techno-economic analysis results.

Procedures	Category	Specification	Cost ($)	Total cost ($)	Notes	Ref.
Solar to ammonia	Electrolyzer	0.2*0.2 m^2	800	1174.7	The price of electrolyzer is assumed to be 20000 $ m^{-2}	2
	Catalyst	0.4 *10^{-3} kg (Estimated based on the experiment.)	80		Catalyst cost is assumed to be 5% of the electrolyzer cost.	3
	Product separation	The purpose is to separate the ammonia from electrolyte.	123.7		The product separation cost is assumed to be 10% of the NH_3 revenue price in 2023	14
	Solar panels	2*10^{-4} m^2	171		The cost of solar panels is based on the price that we bought.	15
Electricity to ammonia	Electrolyzer	0.2*0.2 m^2	800	1311.7	The price of electrolyzer is assumed to be 20000 $ m^{-2}	2
	Catalyst	0.4 *10^{-3} kg (Estimated based on the experiment)	80		Catalyst cost is assumed to be 5% of the electrolyzer cost.	3
	Product separation	The purpose is to separate the ammonia	123.7		The product separation cost is	14

		from electrolyte.			assumed to be 10% of the NH_3 revenue price in 2023.	
	Electricity	3.08×10*4 kWh (The electricity is estimated based on the electrochemically produce one ton of ammonia.)	308		The electricity price is reduced to 1 cent kWh^{-1}	
NH_3 revenue	Revenue	-	-	1237	-	4

Note: The cost of producing ammonia from solar energy is relatively low, but due to the limitations of solar energy supply itself, such as working only when it is sunny, leading to unstable production and other problems. If these problems can be solved in the future, solar-driven ammonia production will be an economical, environmentally friendly, and green path.

Movie S1 (separate file). The formation process of amorphous/crystalline nanoparticles by laser irradiation in water.

References

[1] Chen, G. *et al.* A long-range disordering RuO_2 catalyst for highly efficient acidic oxygen evolution electrocatalysis. *Angew. Chem. Int. Ed.*, e202411603 (2024).

[2] Krishnan, S. *et al.* Present and future cost of alkaline and PEM electrolyser stacks. *Int. J. Hydrogen Energy* **48**, 32313-32330 (2023).

[3] Fan, K. *et al.* Active hydrogen boosts electrochemical nitrate reduction to ammonia. *Nat. Commun.* **13**, 7958 (2022).

[4] Schnitkey, G., N. Paulson, C. Zulauf, and J. Baltz. Fertilizer Prices and Company Profits Going into Spring 2023. *farmdoc daily* **13**, 36 (2023).

[5] Zhang, S. *et al.* Fe/Cu diatomic catalysts for electrochemical nitrate reduction to ammonia. *Nat. Commun.* **14**, 3634 (2023).

[6] Gao, Q. *et al.* Breaking adsorption-energy scaling limitations of electrocatalytic nitrate reduction on intermetallic CuPd nanocubes by machine-learned insights. *Nat. Commun.* **13**, 2338 (2022).

[7] Chen, G.-F. *et al.* Electrochemical reduction of nitrate to ammonia via direct eight-electron transfer using a copper–molecular solid catalyst. *Nat. Energy* **5**, 605-613 (2020).

[8] Fang, J. Y. *et al.* Ampere-level current density ammonia electrochemical synthesis using CuCo nanosheets simulating nitrite reductase bifunctional nature. *Nat. Commun.* **13**, 7899 (2022).

[9] Liao, W. *et al.* Sustainable conversion of alkaline nitrate to ammonia at activities greater than 2 A cm(-2). *Nat Commun* **15**, 1264 (2024).

[10] Xu, W. *et al.* Metal-Oxo Electronic Tuning via In Situ CO Decoration for Promoting Methane Conversion to Oxygenates over Single-Atom Catalysts. *Angew. Chem. Int. Ed.* **63**, e202315343 (2024).

[11] Chen, F. Y. *et al.* Efficient conversion of low-concentration nitrate sources into ammonia on a Ru-dispersed Cu nanowire electrocatalyst. *Nat. Nanotechnol.* **17**, 759–767 (2022).

[12] Zhang, R. *et al.* Efficient ammonia electrosynthesis and energy conversion through a Zn-nitrate battery by iron doping engineered nickel phosphide catalyst. *Adv. Energy Mater.* **12**, 2103872 (2022).

[13] Han, S. *et al.* Ultralow overpotential nitrate reduction to ammonia via a three-step relay mechanism. *Nat. Catal.* **6**, 402–414 (2023).

[14] Duan, Y. *et al.* Process simulation and evaluation for NH_3/CO_2 separation from melamine tail gas with protic ionic liquids. *Sep. Purif. Technol.* **288** (2022).

[15] Frank, U. *et al.* Determination of 2D Particle Size Distributions in Plasmonic Nanoparticle Colloids via Analytical Ultracentrifugation: Application to Gold Bipyramids. *ACS Nano* **17**, 5785-5798 (2023).
